# Supplementary material for: Bifidobacterium Is Enriched in Gut Microbiome of Kashmiri Women with Polycystic Ovary Syndrome
Source: Genes (Basel). 2022 Feb 18;13(2):379. doi: 10.3390/genes13020379 (PMC8871983; doi:10.3390/genes13020379)
Supplement: Supplementary file 1 [file genes-13-00379-s001.zip › FigureS1A.html]

Javascript must be enabled to view this page.

magnitude

 99.9990526315788

 99.8486842105262

 48.4497368421052

 34.4764736842105

 31.6099473684211

 2.65263157894737E-02

 2.65263157894737E-02

 .001

 2.55263157894737E-02

 3.57642105263158

 2.10526315789474E-04

 2.10526315789474E-04

 .494631578947368

 .159157894736842

 0

 .193210526315789

 .142263157894737

 2.10526315789474E-04

 1.05263157894737E-04

 1.05263157894737E-04

 2.82631578947368E-02

 3.68421052631579E-04

 1.05263157894737E-04

 2.54736842105263E-02

 1.63157894736842E-03

 5.26315789473684E-05

 6.31578947368421E-04

 2.03968421052631

 2.63157894736842E-04

 5.26315789473684E-05

 1.57894736842105E-04

 .209684210526316

 1.57894736842105E-04

 1.05263157894737E-04

 2.10526315789474E-03

 2.63157894736842E-04

 .206315789473684

 1.37368421052632E-02

 7.89473684210526E-04

 5.26315789473684E-05

 2.84210526315789E-03

 2.46315789473684E-02

 1.05263157894737E-04

 8.94736842105263E-04

 3.15789473684211E-04

 3.32631578947368E-02

 5.26315789473684E-05

 6.54736842105263E-02

 .137842105263158

 3.77368421052632E-02

 .005

 1.57894736842105E-04

 7.94736842105263E-03

 1.57894736842105E-04

 5.26315789473684E-05

 3.68421052631579E-04

 2.10526315789474E-04

 8.21052631578947E-03

 2.26315789473684E-03

 5.31578947368421E-03

 1.63157894736842E-03

 5.57894736842105E-03

 4.21052631578947E-04

 1.48421052631579E-02

 1.46315789473684E-02

 3.15789473684211E-04

 1.23573684210526

 2.63157894736842E-04

 1.05263157894737E-04

 1.57894736842105E-04

 1.67894736842105E-02

 5.63157894736842E-03

 1.11578947368421E-02

 .322578947368421

 1.44210526315789E-02

 .308157894736842

 7.89999999999999E-02

 3.68421052631579E-04

 5.76315789473684E-02

 1.15789473684211E-03

 6.8421052631579E-04

 1.01052631578947E-02

 3.26315789473684E-03

 3.68421052631579E-04

 5.42105263157895E-03

 .262315789473684

 .115736842105263

 7.83684210526316E-02

 6.82105263157895E-02

 4.86315789473684E-02

 4.78421052631579E-02

 7.89473684210526E-04

 5.78947368421053E-04

 5.78947368421053E-04

 3.94736842105263E-03

 2.10526315789474E-04

 3.73684210526316E-03

 .279315789473684

 .279315789473684

 4.36794736842106

 .043578947368421

 1.56842105263158E-02

 4.63157894736842E-03

 2.32631578947368E-02

 5.26315789473684E-05

 5.26315789473684E-05

 9.47368421052632E-04

 6.8421052631579E-04

 2.63157894736842E-04

 1.01052631578947E-02

 9.52631578947368E-03

 5.78947368421053E-04

 9.47368421052632E-03

 2.78947368421053E-03

 6.68421052631579E-03

 3.37042105263158

 2.41963157894737

 .922842105263158

 5.26315789473684E-05

 1.05263157894737E-04

 2.77894736842105E-02

 .210526315789474

 7.94736842105263E-03

 .112578947368421

 .09

 .353157894736842

 .352842105263158

 3.15789473684211E-04

 1.14210526315789E-02

 1.14210526315789E-02

 .171263157894737

 2.85263157894737E-02

 1.34736842105263E-02

 .129263157894737

 2.26315789473684E-03

 2.26315789473684E-03

 3.53684210526315E-02

 1.54210526315789E-02

 1.94736842105263E-02

 4.73684210526316E-04

 6.59473684210526E-02

 6.59473684210526E-02

 8.34210526315791E-02

 2.17368421052632E-02

 9.89473684210526E-03

 1.05263157894737E-03

 2.47368421052632E-03

 1.67368421052632E-02

 9.78947368421053E-03

 2.17368421052632E-02

 12.23

 7.92631578947368E-02

 1.55789473684211E-02

 2.11052631578947E-02

 .042578947368421

 4.41052631578947E-02

 4.39473684210526E-02

 1.57894736842105E-04

 7.61173684210527

 3.61489473684211

 3.99684210526316

 2.06373684210527

 .241473684210526

 .026

 1.79626315789474

 2.41752631578947

 1.46315789473684E-02

 .666842105263158

 .166157894736842

 3.73684210526316E-03

 1.56615789473684

 1.36315789473684E-02

 1.36315789473684E-02

 9.46494736842106

 2.46136842105263

 .419894736842105

 1.78947368421053E-03

 .042

 8.15263157894737E-02

 5.05263157894737E-03

 .135315789473684

 1.77578947368421

 .243842105263158

 .110684210526316

 .133157894736842

 1.73684210526316E-03

 1.73684210526316E-03

 .843789473684211

 .091

 .438368421052632

 .314421052631579

 .291631578947369

 .142789473684211

 .148842105263158

 9.84210526315789E-03

 8.94736842105263E-03

 8.94736842105263E-04

 .941368421052632

 .302526315789474

 .638842105263158

 .193736842105263

 .161

 3.27368421052632E-02

 .185263157894737

 .185263157894737

 2.98789473684211

 .983421052631579

 2.00447368421053

 1.12873684210526

 .665526315789474

 2.36842105263158E-03

 .460842105263158

 1.33684210526316E-02

 1.33684210526316E-02

 1.29473684210526E-02

 1.29473684210526E-02

 2.92105263157895E-02

 2.92105263157895E-02

 .120210526315789

 .120210526315789

 2.89473684210526E-03

 2.89473684210526E-03

 2.89473684210526E-03

 .36778947368421

 .363263157894737

 2.10526315789474E-04

 .005

 .358052631578947

 2.63157894736842E-04

 2.63157894736842E-04

 7.36842105263158E-04

 7.36842105263158E-04

 3.47368421052632E-03

 3.47368421052632E-03

 5.26315789473684E-05

 5.26315789473684E-05

 .139526315789474

 6.05263157894737E-03

 2.31578947368421E-03

 8.42105263157895E-04

 2.89473684210526E-03

 3.22105263157894E-02

 4.21052631578947E-04

 2.27894736842105E-02

 8.89473684210526E-03

 1.05263157894737E-04

 5.10526315789474E-03

 5.10526315789474E-03

 2.78947368421052E-03

 2.57894736842105E-03

 2.10526315789474E-04

 1.12631578947368E-02

 1.12631578947368E-02

 7.31578947368421E-03

 7.31578947368421E-03

 7.47894736842105E-02

 7.47894736842105E-02

 9.05263157894737E-02

 9.04736842105263E-02

 6.31578947368421E-04

 8.98421052631579E-02

 5.26315789473684E-05

 5.26315789473684E-05

 7.15789473684211E-03

 7.15789473684211E-03

 7.15789473684211E-03

 2.10526315789474E-03

 2.10526315789474E-03

 2.10526315789474E-03

 6.84210526315789E-04

 6.84210526315789E-04

 1.05263157894737E-04

 1.57894736842105E-04

 4.21052631578947E-04

 8.26315789473684E-03

 8.26315789473684E-03

 8.26315789473684E-03

 1.32515789473684

 1.32515789473684

 1.32515789473684

 2.50105263157894

 2.50105263157894

 2.23684210526316E-02

 1.69473684210526E-02

 5.42105263157895E-03

 .251157894736842

 4.42105263157895E-03

 3.68421052631579E-04

 1.57894736842105E-04

 .246210526315789

 1.56536842105263

 1.32473684210526

 .104526315789474

 5.94736842105263E-02

 7.66315789473684E-02

 2.63157894736842E-03

 2.63157894736842E-03

 3.92631578947369E-02

 1.66842105263158E-02

 2.25789473684211E-02

 4.75789473684211E-02

 4.75789473684211E-02

 .547421052631579

 1.05263157894737E-04

 1.31578947368421E-03

 5.26315789473684E-05

 9.14736842105263E-02

 .08

 .374473684210526

 2.52631578947368E-02

 2.52631578947368E-02

 8.84736842105264E-02

 1.68947368421053E-02

 3.36842105263158E-03

 3.36842105263158E-03

 4.73684210526316E-04

 4.73684210526316E-04

 5.21052631578948E-03

 1.42105263157895E-03

 3.78947368421053E-03

 3.10526315789474E-03

 3.10526315789474E-03

 0

 0

 2.94736842105264E-03

 4.21052631578947E-04

 3.68421052631579E-04

 9.47368421052632E-04

 5.26315789473684E-05

 1.15789473684211E-03

 1.78947368421053E-03

 1.78947368421053E-03

 3.95263157894737E-02

 3.95263157894737E-02

 3.15789473684211E-04

 3.92105263157895E-02

 2.68421052631579E-02

 2.68421052631579E-02

 2.68421052631579E-02

 5.21052631578947E-03

 5.21052631578947E-03

 5.21052631578947E-03

 8.66315789473684E-02

 8.04210526315789E-02

 8.04210526315789E-02

 8.04210526315789E-02

 6.15789473684211E-03

 6.15789473684211E-03

 6.15789473684211E-03

 5.26315789473684E-05

 5.26315789473684E-05

 5.26315789473684E-05

 1.23157894736842E-02

 7.21052631578947E-03

 6.94736842105263E-03

 6.94736842105263E-03

 1.57894736842105E-04

 1.57894736842105E-04

 1.05263157894737E-04

 1.05263157894737E-04

 5.10526315789474E-03

 5.10526315789474E-03

 6.8421052631579E-04

 1.05263157894737E-03

 3.36842105263158E-03

 .178052631578947

 .178052631578947

 .178052631578947

 .178052631578947

 10.2943684210526

 4.51342105263158

 4.35894736842105

 1.94736842105264E-03

 1.10526315789474E-03

 8.42105263157895E-04

 7.36842105263158E-04

 7.36842105263158E-04

 8.45263157894736E-02

 4.21052631578947E-04

 7.94210526315789E-02

 1.05263157894737E-04

 5.26315789473684E-05

 1.57894736842105E-04

 5.26315789473684E-05

 4.31578947368421E-03

 4.19178947368421

 5.26315789473684E-05

 5.06315789473684E-02

 5.42105263157895E-03

 2.10526315789474E-04

 5.26315789473684E-05

 5.26315789473684E-05

 2.21052631578947E-03

 3.15789473684211E-04

 5.26315789473684E-05

 4.21052631578947E-04

 1.57894736842105E-04

 1.05263157894737E-03

 0

 5.26315789473684E-05

 1.89473684210526E-03

 1.57894736842105E-04

 5.26315789473684E-05

 5.78947368421053E-04

 1.57894736842105E-04

 3.43384210526316

 5.26315789473684E-05

 3.85263157894737E-02

 6.67894736842105E-02

 1.05263157894737E-04

 1.73684210526316E-03

 1.05263157894737E-04

 .286578947368421

 5.36842105263158E-03

 5.26315789473684E-04

 5.26315789473684E-05

 3.15789473684211E-04

 .294263157894737

 4.36842105263158E-03

 2.63157894736842E-04

 4.10526315789474E-03

 8.52631578947369E-03

 5.78947368421053E-04

 7.68421052631579E-03

 2.63157894736842E-04

 1.21052631578947E-03

 8.94736842105263E-04

 3.15789473684211E-04

 5.26315789473684E-04

 5.26315789473684E-04

 9.42105263157895E-03

 1.26315789473684E-03

 8.15789473684211E-03

 2.10526315789474E-04

 2.10526315789474E-04

 4.28421052631579E-02

 4.28421052631579E-02

 1.28421052631579E-02

 1.57894736842105E-04

 4.21052631578947E-04

 1.22631578947368E-02

 4.27368421052632E-02

 7.89473684210526E-04

 7.89473684210526E-04

 7.89473684210527E-03

 1.57894736842105E-04

 .001

 1.42105263157895E-03

 0

 5.31578947368421E-03

 3.03157894736842E-02

 5.26315789473684E-05

 5.26315789473684E-05

 5.26315789473684E-05

 4.21052631578947E-04

 1.57894736842105E-04

 3.15789473684211E-04

 7.8421052631579E-03

 5.26315789473684E-05

 2.13684210526316E-02

 3.73684210526316E-03

 3.73684210526316E-03

 4.73684210526315E-03

 4.21052631578947E-03

 4.21052631578947E-03

 4.73684210526315E-04

 4.21052631578947E-04

 5.26315789473684E-05

 5.26315789473684E-05

 5.26315789473684E-05

 1.24736842105263E-02

 1.21052631578948E-03

 1.15789473684211E-03

 5.26315789473684E-05

 4.73684210526316E-04

 2.10526315789474E-04

 2.63157894736842E-04

 .008

 1.05263157894737E-04

 5.26315789473684E-05

 2.63157894736842E-04

 1.05263157894737E-04

 3.15789473684211E-04

 1.57894736842105E-04

 .007

 2.57894736842105E-03

 2.57894736842105E-03

 2.10526315789474E-04

 2.10526315789474E-04

 1.99473684210526E-02

 3.78947368421053E-03

 2.21052631578947E-03

 1.42105263157895E-03

 1.05263157894737E-04

 5.26315789473684E-05

 1.57894736842105E-04

 1.57894736842105E-04

 5.05263157894737E-03

 2.89473684210526E-03

 2.15789473684211E-03

 2.63157894736842E-04

 5.26315789473684E-05

 5.26315789473684E-05

 5.26315789473684E-05

 5.26315789473684E-05

 5.26315789473684E-05

 9.94736842105263E-03

 5.26315789473684E-04

 7.36842105263158E-04

 8.42105263157895E-03

 2.63157894736842E-04

 2.10526315789474E-04

 2.10526315789474E-04

 5.26315789473684E-04

 5.26315789473684E-04

 6.36842105263158E-03

 6.36842105263158E-03

 6.36842105263158E-03

 3.31578947368421E-03

 3.31578947368421E-03

 3.31578947368421E-03

 0

 0

 0

 6.48947368421053E-02

 6.48947368421053E-02

 6.48947368421053E-02

 5.44478947368421

 8.32631578947369E-02

 8.31578947368421E-02

 7.89473684210526E-04

 8.23684210526316E-02

 0

 0

 1.05263157894737E-04

 1.05263157894737E-04

 4.18421052631578E-02

 3.21052631578947E-03

 3.21052631578947E-03

 2.21052631578947E-03

 1.26315789473684E-03

 5.26315789473684E-05

 8.94736842105263E-04

 3.64210526315789E-02

 3.64210526315789E-02

 4.85789473684211E-02

 3.77368421052632E-02

 0

 5.26315789473684E-05

 1.15789473684211E-03

 9.78947368421053E-03

 6.73684210526316E-03

 5.26315789473684E-05

 5.26315789473684E-05

 5.84210526315789E-03

 1.89473684210526E-03

 1.21578947368421E-02

 8.57894736842105E-03

 8.57894736842105E-03

 1.31578947368421E-03

 1.31578947368421E-03

 9.47368421052632E-04

 3.15789473684211E-04

 5.26315789473684E-05

 5.78947368421053E-04

 .034421052631579

 1.10526315789474E-03

 1.10526315789474E-03

 1.13684210526316E-02

 1.57894736842105E-04

 1.12105263157895E-02

 2.36842105263158E-03

 2.36842105263158E-03

 4.21052631578947E-04

 4.21052631578947E-04

 1.91578947368421E-02

 1.28421052631579E-02

 3.84210526315789E-03

 1.05263157894737E-03

 1.05263157894737E-04

 .001

 2.10526315789474E-04

 1.05263157894737E-04

 4.98331578947368

 4.94694736842105

 1.15789473684211E-03

 5.26315789473684E-05

 3.73684210526316E-03

 1.15789473684211E-03

 1.05263157894737E-03

 8.36842105263158E-03

 1.15789473684211E-02

 .283157894736842

 1.57894736842105E-03

 1.05263157894737E-04

 2.42105263157895E-03

 1.05263157894737E-04

 8.94736842105263E-04

 1.63684210526316E-02

 1.89473684210526E-03

 1.05263157894737E-04

 2.05789473684211E-02

 1.57894736842105E-03

 1.57894736842105E-04

 5.26315789473684E-05

 5.26315789473684E-05

 6.8421052631579E-04

 2.10526315789474E-04

 .121052631578947

 2.10526315789474E-04

 4.73684210526316E-04

 7.36842105263158E-04

 1.57894736842105E-04

 1.15789473684211E-03

 1.63157894736842E-03

 1.05263157894737E-04

 4.28915789473684

 4.73684210526316E-04

 5.26315789473684E-05

 6.31578947368421E-04

 3.10526315789474E-03

 1.57894736842105E-04

 5.26315789473684E-05

 1.26315789473684E-03

 1.22631578947368E-02

 7.68421052631579E-03

 2.63157894736842E-04

 .149263157894737

 1.53684210526316E-02

 1.47368421052632E-03

 3.84210526315789E-03

 3.68421052631579E-04

 3.68421052631579E-04

 1.57894736842105E-04

 5.26315789473684E-05

 9.10526315789474E-03

 .021

 .021

 .194

 6.8421052631579E-03

 4.73684210526316E-04

 5.47368421052632E-03

 1.05263157894737E-04

 2.10526315789474E-04

 5.78947368421053E-04

 .187157894736842

 4.21052631578947E-04

 6.31578947368421E-04

 5.78947368421053E-04

 4.68421052631579E-03

 5.26315789473684E-05

 5.26315789473684E-04

 3.26315789473684E-03

 2.63157894736842E-04

 5.78947368421053E-04

 7.36842105263158E-04

 .002

 1.05263157894737E-04

 1.05263157894737E-04

 1.05263157894737E-04

 9.47368421052632E-04

 0

 7.36842105263158E-04

 0

 2.52631578947368E-03

 5.26315789473684E-05

 4.73684210526316E-04

 5.26315789473684E-05

 2.57894736842105E-03

 1.52631578947368E-03

 5.26315789473684E-05

 5.26315789473684E-05

 3.45789473684211E-02

 2.52631578947368E-03

 4.62105263157895E-02

 8.07894736842105E-02

 5.93684210526316E-02

 5.93684210526316E-02

 5.93684210526316E-02

 4.68421052631579E-03

 4.68421052631579E-03

 4.68421052631579E-03

 4.21052631578947E-04

 8.94736842105263E-04

 1.63157894736842E-03

 1.73684210526316E-03

 4.18421052631579E-02

 4.18421052631579E-02

 4.18421052631579E-02

 4.08421052631579E-02

 1.05263157894737E-04

 8.94736842105263E-04

 .155526315789473

 .155526315789473

 .155526315789473

 2.90526315789474E-02

 .126473684210526

 8.79473684210526E-02

 8.79473684210526E-02

 8.79473684210526E-02

 8.79473684210526E-02

 4.61578947368421E-02

 4.61578947368421E-02

 4.61578947368421E-02

 4.61578947368421E-02

 3.47568421052631

 3.47568421052631

 2.34810526315789

 2.10526315789474E-04

 2.10526315789474E-04

 3.40526315789474E-02

 3.68421052631579E-04

 4.73684210526316E-04

 3.32105263157895E-02

 1.40642105263157

 1.06315789473684E-02

 1.39410526315789

 1.68421052631579E-03

 .902947368421052

 .696473684210526

 .173210526315789

 4.73684210526316E-04

 3.27894736842105E-02

 4.15789473684211E-03

 4.15789473684211E-03

 3.15789473684211E-04

 3.15789473684211E-04

 1.08073684210526

 1.07621052631579

 8.47368421052632E-02

 .991473684210526

 4.10526315789474E-03

 3.68421052631579E-03

 4.21052631578947E-04

 3.68421052631579E-04

 2.63157894736842E-04

 1.05263157894737E-04

 5.26315789473684E-05

 5.26315789473684E-05

 4.68421052631579E-02

 4.68421052631579E-02

 4.68421052631579E-02

 .203210526315789

 .203210526315789

 .203210526315789

 .203210526315789

 .203210526315789

 7.74736842105263E-02

 7.74736842105263E-02

 4.14210526315789E-02

 4.14210526315789E-02

 3.43157894736842E-02

 5.26315789473684E-05

 2.56315789473684E-02

 5.26315789473684E-05

 6.57894736842105E-03

 1.26315789473684E-03

 7.36842105263158E-04

 7.05263157894737E-03

 4.68421052631579E-03

 2.36842105263158E-03

 5.26315789473684E-05

 5.26315789473684E-05

 .017

 .017

 .017

 .017

 1.48421052631579E-02

 1.48421052631579E-02

 1.47368421052632E-02

 8.94736842105263E-04

 5.26315789473684E-05

 1.05263157894737E-04

 8.42105263157895E-04

 1.78947368421053E-03

 3.68421052631579E-04

 1.52631578947368E-03

 5.26315789473684E-05

 9.10526315789474E-03

 1.05263157894737E-04

 1.05263157894737E-04

 0

 0

 0

 0

 4.21052631578947E-03

 4.21052631578947E-03

 4.21052631578947E-03

 4.21052631578947E-03

 8.71136842105263

 1.6528947368421

 5.14736842105263E-02

 1.03684210526316E-02

 2.10526315789474E-04

 5.26315789473684E-05

 5.26315789473684E-05

 1.05263157894737E-04

 9.47368421052632E-04

 9.47368421052632E-04

 2.10526315789474E-04

 5.26315789473684E-05

 5.26315789473684E-05

 1.05263157894737E-04

 5.26315789473684E-05

 5.26315789473684E-05

 6.94736842105263E-03

 1.57894736842105E-04

 5.26315789473684E-05

 4.89473684210526E-03

 8.42105263157895E-04

 0

 .001

 1.57894736842105E-04

 1.57894736842105E-04

 1.84210526315789E-03

 1.84210526315789E-03

 2.97894736842105E-02

 1.00526315789474E-02

 7.36842105263158E-03

 4.73684210526316E-04

 2.21052631578947E-03

 2.63157894736842E-04

 2.63157894736842E-04

 1.05263157894737E-04

 1.05263157894737E-04

 7.36842105263157E-04

 6.84210526315789E-04

 5.26315789473684E-05

 4.57894736842106E-03

 5.26315789473684E-05

 5.26315789473684E-05

 4.47368421052632E-03

 3.68421052631579E-03

 2.31578947368421E-03

 1.36842105263158E-03

 1.57894736842105E-04

 1.57894736842105E-04

 4.73684210526316E-03

 4.73684210526316E-03

 0

 0

 5.47368421052632E-03

 5.47368421052632E-03

 1.13157894736842E-02

 1.13157894736842E-02

 1.13157894736842E-02

 .127578947368421

 9.15789473684211E-03

 1.57894736842105E-03

 2.10526315789474E-04

 1.57894736842105E-04

 5.26315789473684E-04

 2.10526315789474E-04

 4.73684210526316E-04

 3.52631578947369E-03

 1.36842105263158E-03

 5.26315789473684E-05

 2.10526315789474E-03

 1.73684210526316E-03

 2.10526315789474E-04

 1.57894736842105E-04

 2.63157894736842E-04

 1.05263157894737E-04

 .001

 2.63157894736842E-04

 2.10526315789474E-04

 5.26315789473684E-05

 2.10526315789474E-04

 2.10526315789474E-04

 1.84210526315789E-03

 1.84210526315789E-03

 .085421052631579

 5.26315789473684E-05

 5.26315789473684E-05

 1.36842105263158E-03

 8.42105263157895E-04

 5.26315789473684E-04

 8.36842105263158E-02

 6.89473684210526E-03

 2.57894736842105E-03

 1.05263157894737E-04

 .001

 5.26315789473684E-05

 7.30526315789474E-02

 3.15789473684211E-04

 1.05263157894737E-04

 2.10526315789474E-04

 1.05263157894737E-04

 1.05263157894737E-04

 5.26315789473684E-05

 5.26315789473684E-05

 1.10526315789474E-03

 1.10526315789474E-03

 5.26315789473684E-05

 8.94736842105263E-04

 1.57894736842105E-04

 3.68421052631579E-04

 5.26315789473684E-05

 5.26315789473684E-05

 3.15789473684211E-04

 3.15789473684211E-04

 5.26315789473684E-03

 5.26315789473684E-04

 5.26315789473684E-04

 8.42105263157894E-04

 5.26315789473684E-05

 7.89473684210526E-04

 .002

 .002

 2.10526315789474E-04

 2.10526315789474E-04

 5.26315789473684E-05

 5.26315789473684E-05

 1.63157894736842E-03

 1.63157894736842E-03

 4.26315789473684E-03

 1.36842105263158E-03

 6.8421052631579E-04

 6.8421052631579E-04

 1.42105263157894E-03

 1.57894736842105E-04

 1.26315789473684E-03

 1.36842105263158E-03

 1.36842105263158E-03

 1.05263157894737E-04

 1.05263157894737E-04

 4.57894736842106E-03

 2.68421052631579E-03

 4.21052631578947E-04

 1.57894736842105E-04

 2.10526315789474E-04

 1.05263157894737E-04

 1.57894736842105E-04

 1.57894736842105E-04

 1.47368421052632E-03

 6.84210526315789E-04

 4.21052631578947E-04

 2.63157894736842E-04

 5.78947368421052E-04

 1.57894736842105E-04

 4.21052631578947E-04

 0

 0

 6.31578947368421E-04

 6.31578947368421E-04

 3.21052631578948E-03

 3.21052631578948E-03

 1.05263157894737E-03

 2.15789473684211E-03

 3.1578947368421E-04

 3.1578947368421E-04

 5.26315789473684E-05

 1.05263157894737E-04

 1.57894736842105E-04

 2.89473684210526E-03

 2.84210526315789E-03

 1.05263157894737E-04

 7.89473684210526E-04

 1.94736842105263E-03

 5.26315789473684E-05

 5.26315789473684E-05

 1.57894736842105E-04

 1.57894736842105E-04

 1.57894736842105E-04

 1.07368421052632E-02

 1.07368421052632E-02

 1.07368421052632E-02

 1.35994736842105

 1.3548947368421

 3.47368421052632E-03

 1.78947368421053E-03

 8.94736842105263E-04

 7.89473684210526E-04

 2.63157894736842E-04

 1.57894736842105E-04

 5.26315789473684E-05

 5.26315789473684E-05

 1.57894736842105E-04

 1.05263157894737E-04

 5.26315789473684E-05

 1.22736842105263

 5.26315789473684E-05

 3.68421052631579E-04

 9.73684210526316E-03

 .206473684210526

 4.68421052631579E-03

 5.26315789473684E-04

 1.57894736842105E-04

 1.03157894736842E-02

 5.26315789473684E-05

 2.57894736842105E-03

 5.26315789473684E-05

 7.89473684210526E-04

 5.26315789473684E-05

 3.21052631578947E-03

 6.84210526315789E-04

 .987631578947368

 1.51578947368421E-02

 5.26315789473684E-05

 1.51052631578947E-02

 5.26315789473684E-05

 5.26315789473684E-05

 .083578947368421

 8.23157894736842E-02

 1.05263157894737E-04

 1.15789473684211E-03

 5.26315789473684E-05

 5.26315789473684E-05

 2.94736842105264E-03

 1.57894736842105E-04

 1.47368421052632E-03

 1.31578947368421E-03

 6.26315789473684E-03

 6.05263157894737E-03

 2.10526315789474E-04

 5.26315789473684E-04

 5.26315789473684E-04

 1.50526315789474E-02

 1.50526315789474E-02

 3.10526315789474E-03

 1.47368421052632E-03

 5.26315789473684E-05

 1.42105263157895E-03

 1.47368421052632E-03

 1.47368421052632E-03

 1.57894736842105E-04

 1.57894736842105E-04

 1.94736842105263E-03

 1.94736842105263E-03

 1.94736842105263E-03

 2.15263157894737E-02

 2.15263157894737E-02

 1.17368421052631E-02

 9.21052631578947E-03

 2.52631578947368E-03

 1.36842105263158E-03

 1.57894736842105E-04

 5.26315789473684E-05

 1.15789473684211E-03

 2.47368421052632E-03

 1.57894736842105E-04

 2.15789473684211E-03

 1.57894736842105E-04

 5.26315789473684E-05

 5.26315789473684E-05

 0

 2.21052631578947E-03

 2.10526315789474E-04

 4.21052631578947E-04

 1.57894736842105E-03

 3.68421052631579E-03

 3.68421052631579E-03

 4.41578947368421E-02

 9.63157894736842E-03

 7.68421052631579E-03

 7.68421052631579E-03

 1.94736842105263E-03

 1.84210526315789E-03

 1.05263157894737E-04

 3.43684210526316E-02

 3.43684210526316E-02

 5.26315789473684E-05

 3.43157894736842E-02

 1.57894736842105E-04

 1.57894736842105E-04

 1.57894736842105E-04

 2.82631578947368E-02

 1.89473684210526E-03

 1.73684210526316E-03

 1.73684210526316E-03

 1.57894736842105E-04

 1.57894736842105E-04

 2.60526315789474E-02

 1.89473684210526E-03

 5.78947368421053E-04

 1.31578947368421E-03

 .003

 8.42105263157895E-04

 2.63157894736842E-04

 1.57894736842105E-04

 0

 1.57894736842105E-04

 1.57894736842105E-04

 1.42105263157895E-03

 2.73684210526316E-03

 1.31578947368421E-03

 5.26315789473684E-05

 5.26315789473684E-05

 1.31578947368421E-03

 1.44736842105263E-02

 1.57894736842105E-04

 3.15789473684211E-04

 1.05263157894737E-04

 1.05263157894737E-04

 1.05263157894737E-04

 5.26315789473684E-05

 7.36842105263158E-04

 2.10526315789474E-04

 5.26315789473684E-05

 5.47368421052632E-03

 1.89473684210526E-03

 5.26315789473684E-05

 5.26315789473684E-05

 2.63157894736842E-04

 4.89473684210526E-03

 3.68421052631579E-04

 5.26315789473684E-05

 1.05263157894737E-04

 2.10526315789474E-04

 3.57894736842105E-03

 3.57894736842105E-03

 3.15789473684211E-04

 3.15789473684211E-04

 3.15789473684211E-04

 6.84210526315789E-04

 6.84210526315789E-04

 6.84210526315789E-04

 5.26315789473684E-05

 6.31578947368421E-04

 1.92631578947368E-02

 1.92631578947368E-02

 1.92631578947368E-02

 1.92631578947368E-02

 .763999999999999

 .704473684210526

 .178105263157895

 1.73684210526316E-03

 1.57894736842105E-04

 6.31578947368421E-04

 1.05263157894737E-04

 5.26315789473684E-05

 4.73684210526316E-04

 3.15789473684211E-04

 2.10526315789473E-04

 5.26315789473684E-05

 1.57894736842105E-04

 1.63157894736842E-03

 2.63157894736842E-04

 2.63157894736842E-04

 1.10526315789474E-03

 2.05263157894737E-03

 2.05263157894737E-03

 2.24736842105264E-02

 1.05263157894737E-04

 5.15789473684211E-03

 1.57368421052632E-02

 1.47368421052632E-03

 3.15789473684211E-04

 3.15789473684211E-04

 1.21052631578948E-03

 1.10526315789474E-03

 1.05263157894737E-04

 2.63157894736842E-04

 2.63157894736842E-04

 2.10526315789474E-04

 2.10526315789474E-04

 4.21052631578947E-04

 5.26315789473684E-05

 3.68421052631579E-04

 7.36842105263158E-04

 5.78947368421053E-04

 1.57894736842105E-04

 5.26315789473684E-05

 5.26315789473684E-05

 5.33684210526316E-02

 5.33684210526316E-02

 .041

 5.26315789473684E-04

 4.04210526315789E-02

 5.26315789473684E-05

 1.57894736842105E-04

 1.57894736842105E-04

 1.57894736842105E-04

 1.57894736842105E-04

 1.05263157894737E-03

 1.05263157894737E-03

 2.63157894736842E-04

 2.63157894736842E-04

 2.63157894736842E-04

 2.63157894736842E-04

 5.26315789473684E-05

 5.26315789473684E-05

 .048

 .048

 2.47368421052631E-03

 1.57894736842105E-04

 1.94736842105263E-03

 5.26315789473684E-05

 3.15789473684211E-04

 0

 0

 8.21052631578947E-03

 5.36842105263158E-03

 2.52631578947368E-03

 5.26315789473684E-05

 8.42105263157895E-04

 2.10526315789474E-04

 1.73684210526316E-03

 8.94736842105263E-04

 8.94736842105263E-04

 1.89473684210526E-03

 1.89473684210526E-03

 5.26315789473684E-05

 5.26315789473684E-05

 1.77894736842105E-02

 1.73684210526316E-03

 3.68421052631579E-04

 1.36842105263158E-03

 6.63157894736842E-03

 4.42105263157895E-03

 2.21052631578947E-03

 .003

 5.26315789473684E-05

 2.94736842105263E-03

 3.52631578947369E-03

 2.63157894736842E-04

 5.26315789473684E-05

 3.10526315789474E-03

 1.05263157894737E-04

 5.26315789473684E-05

 5.26315789473684E-05

 5.26315789473684E-05

 5.26315789473684E-05

 5.78947368421053E-04

 5.78947368421053E-04

 2.21052631578947E-03

 2.21052631578947E-03

 .475947368421052

 2.10526315789474E-04

 1.05263157894737E-04

 1.05263157894737E-04

 .475157894736841

 3.62105263157895E-02

 4.21052631578947E-04

 .184473684210526

 .254052631578947

 5.78947368421053E-04

 5.78947368421053E-04

 2.44210526315789E-02

 2.44210526315789E-02

 2.44210526315789E-02

 .004

 .004

 2.10526315789474E-04

 2.10526315789474E-04

 1.57894736842105E-04

 5.26315789473684E-05

 5.26315789473684E-05

 5.26315789473684E-05

 1.63157894736842E-03

 1.21052631578947E-03

 2.63157894736842E-04

 1.57894736842105E-04

 5.26315789473684E-05

 5.26315789473684E-05

 1.05263157894737E-04

 1.05263157894737E-04

 1.36842105263158E-03

 1.36842105263158E-03

 4.73684210526316E-04

 4.73684210526316E-04

 .016421052631579

 .016421052631579

 1.31578947368421E-03

 1.21052631578947E-03

 1.05263157894737E-04

 7.36842105263158E-04

 4.21052631578947E-04

 3.15789473684211E-04

 2.42105263157895E-03

 4.21052631578947E-04

 2.63157894736842E-04

 1.73684210526316E-03

 4.73684210526316E-04

 4.73684210526316E-04

 9.47368421052631E-04

 5.26315789473684E-04

 5.26315789473684E-05

 3.68421052631579E-04

 1.68421052631579E-03

 1.68421052631579E-03

 1.05263157894737E-03

 1.05263157894737E-03

 5.26315789473684E-05

 5.26315789473684E-05

 7.73684210526316E-03

 7.73684210526316E-03

 2.42105263157895E-03

 2.42105263157895E-03

 2.42105263157895E-03

 2.42105263157895E-03

 8.21052631578948E-03

 8.21052631578948E-03

 5.05263157894737E-03

 9.47368421052632E-04

 3.47368421052632E-03

 6.31578947368421E-04

 2.84210526315789E-03

 2.31578947368421E-03

 5.26315789473684E-04

 3.15789473684211E-04

 3.15789473684211E-04

 5.26315789473684E-04

 5.26315789473684E-04

 5.26315789473684E-04

 5.26315789473684E-05

 4.73684210526316E-04

 1.05263157894737E-04

 1.05263157894737E-04

 1.05263157894737E-04

 1.05263157894737E-04

 2.78421052631579E-02

 2.78421052631579E-02

 2.78421052631579E-02

 2.78421052631579E-02

 5.15147368421052

 3.73684210526316E-02

 8.52631578947369E-03

 3.31578947368421E-03

 3.15789473684211E-04

 2.63157894736842E-04

 3.68421052631579E-04

 0

 1.05263157894737E-04

 5.26315789473684E-05

 4.73684210526316E-04

 5.26315789473684E-05

 1.68421052631579E-03

 1.52631578947368E-03

 2.63157894736842E-04

 1.26315789473684E-03

 3.31578947368421E-03

 5.26315789473684E-05

 5.26315789473684E-05

 0

 4.73684210526316E-04

 2.73684210526316E-03

 3.68421052631579E-04

 3.68421052631579E-04

 2.88421052631579E-02

 2.10526315789473E-04

 5.26315789473684E-05

 1.57894736842105E-04

 2.86315789473684E-02

 1.57894736842105E-04

 5.26315789473684E-05

 5.26315789473684E-05

 4.21052631578947E-04

 1.15789473684211E-03

 4.73684210526316E-04

 3.05263157894737E-03

 1.57894736842105E-04

 8.42105263157895E-04

 2.63157894736842E-04

 2.63157894736842E-04

 5.26315789473684E-05

 3.57894736842105E-03

 1.05263157894737E-04

 1.05263157894737E-04

 1.05263157894737E-04

 1.05263157894737E-04

 5.26315789473684E-05

 2.10526315789474E-04

 5.26315789473684E-05

 5.78947368421053E-04

 2.21052631578947E-03

 5.26315789473684E-05

 1.21052631578947E-03

 5.26315789473684E-05

 2.63157894736842E-04

 1.57894736842105E-04

 1.28421052631579E-02

 0

 0

 0

 3.50526315789473E-02

 3.50526315789473E-02

 1.15789473684211E-03

 2.10526315789474E-04

 5.26315789473684E-05

 3.15789473684211E-04

 5.78947368421053E-04

 4.73684210526316E-04

 2.10526315789474E-04

 2.63157894736842E-04

 1.05263157894737E-03

 1.05263157894737E-03

 1.05263157894737E-04

 1.05263157894737E-04

 2.17368421052631E-02

 2.16315789473684E-02

 1.05263157894737E-04

 2.94736842105263E-03

 2.94736842105263E-03

 5.26315789473684E-04

 5.26315789473684E-04

 7.05263157894737E-03

 7.05263157894737E-03

 1.25263157894737E-02

 5.26315789473684E-04

 5.26315789473684E-04

 5.26315789473684E-04

 3.10526315789474E-03

 1.57894736842105E-04

 1.57894736842105E-04

 5.26315789473684E-05

 5.26315789473684E-05

 1.63157894736842E-03

 1.57894736842105E-04

 8.94736842105263E-04

 1.05263157894737E-04

 5.26315789473684E-05

 4.21052631578947E-04

 1.57894736842105E-04

 1.57894736842105E-04

 5.78947368421053E-04

 5.78947368421053E-04

 5.26315789473684E-04

 5.26315789473684E-04

 3.8421052631579E-03

 1.26315789473685E-03

 5.26315789473684E-05

 0

 5.26315789473684E-05

 1.15789473684211E-03

 1.10526315789474E-03

 5.26315789473684E-05

 1.05263157894737E-03

 1.47368421052632E-03

 1.47368421052632E-03

 1.73684210526316E-03

 1.73684210526316E-03

 1.26315789473684E-03

 1.05263157894737E-04

 3.68421052631579E-04

 2.63157894736842E-04

 2.63157894736842E-04

 5.26315789473684E-05

 5.26315789473684E-05

 1.57894736842105E-04

 3.05263157894737E-03

 3.05263157894737E-03

 3.05263157894737E-03

 2.77368421052632E-02

 1.76315789473684E-02

 1.05263157894737E-04

 1.05263157894737E-04

 1.68421052631579E-03

 1.68421052631579E-03

 1.05263157894737E-04

 1.05263157894737E-04

 3.15789473684211E-04

 3.15789473684211E-04

 9.47368421052632E-04

 9.47368421052632E-04

 3.1578947368421E-04

 5.26315789473684E-05

 2.63157894736842E-04

 1.73684210526316E-03

 1.73684210526316E-03

 5.78947368421053E-04

 5.78947368421053E-04

 1.18421052631579E-02

 1.18421052631579E-02

 6.89473684210526E-03

 6.84210526315789E-04

 1.57894736842105E-04

 5.26315789473684E-04

 6.31578947368421E-04

 6.31578947368421E-04

 3.73684210526316E-03

 3.73684210526316E-03

 5.26315789473684E-04

 5.26315789473684E-04

 1.31578947368421E-03

 1.31578947368421E-03

 5.26315789473684E-05

 5.26315789473684E-05

 5.26315789473684E-05

 3.15789473684211E-03

 3.15789473684211E-03

 3.15789473684211E-03

 7.57894736842106E-03

 1.15789473684211E-03

 5.26315789473684E-05

 5.26315789473684E-05

 0

 0

 1.10526315789474E-03

 1.10526315789474E-03

 5.26315789473684E-04

 5.26315789473684E-04

 5.26315789473684E-04

 5.89473684210527E-03

 5.89473684210527E-03

 1.05263157894737E-04

 5.78947368421053E-03

 2.77168421052632

 2.73494736842105

 4.21052631578947E-04

 4.21052631578947E-04

 2.47368421052632E-03

 2.47368421052632E-03

 5.26315789473684E-05

 5.26315789473684E-05

 3.47368421052632E-03

 3.47368421052632E-03

 3.8421052631579E-03

 2.10526315789474E-04

 5.26315789473684E-05

 3.15789473684211E-04

 8.94736842105263E-04

 2.36842105263158E-03

 6.8421052631579E-04

 5.26315789473684E-05

 5.26315789473684E-05

 5.26315789473684E-05

 2.10526315789474E-04

 5.26315789473684E-05

 2.63157894736842E-04

 8.94736842105263E-04

 8.42105263157895E-04

 5.26315789473684E-05

 2.68152631578947

 3.78947368421053E-03

 .981684210526316

 7.89473684210526E-04

 .89478947368421

 .333

 5.26315789473684E-04

 2.42105263157895E-03

 .464526315789474

 3.68421052631579E-04

 2.63157894736842E-04

 1.05263157894737E-04

 1.05263157894737E-04

 1.05263157894737E-04

 3.45789473684211E-02

 3.45789473684211E-02

 6.52631578947369E-03

 3.78947368421053E-03

 2.73684210526316E-03

 3.66842105263157E-02

 1.36315789473684E-02

 1.21052631578947E-03

 1.24210526315789E-02

 1.05263157894737E-02

 9.47368421052632E-04

 9.57894736842105E-03

 1.25263157894737E-02

 1.16842105263158E-02

 8.42105263157895E-04

 5.26315789473684E-05

 5.26315789473684E-05

 5.26315789473684E-05

 .801789473684211

 .801789473684211

 9.68421052631578E-03

 5.26315789473684E-05

 1.57894736842105E-04

 9.47368421052631E-03

 1.05263157894737E-04

 1.05263157894737E-04

 .002

 9.47368421052632E-04

 1.05263157894737E-04

 9.47368421052632E-04

 5.26315789473684E-04

 5.26315789473684E-05

 4.73684210526316E-04

 4.06315789473684E-02

 2.15789473684211E-03

 4.15789473684211E-03

 1.68421052631579E-03

 0

 1.96315789473684E-02

 7.89473684210526E-04

 1.47368421052632E-03

 5.26315789473684E-04

 1.02105263157895E-02

 2.78947368421053E-03

 2.36842105263158E-03

 5.26315789473684E-05

 1.57894736842105E-04

 2.10526315789474E-04

 .587315789473684

 .235526315789474

 8.89473684210526E-03

 .342894736842105

 2.32631578947368E-02

 2.78947368421053E-03

 1.05263157894737E-04

 2.63157894736842E-03

 8.42105263157895E-03

 9.31578947368421E-03

 5.26315789473684E-05

 5.26315789473684E-05

 5.46315789473684E-02

 5.39473684210526E-02

 6.8421052631579E-04

 8.94736842105263E-04

 3.15789473684211E-04

 4.21052631578947E-04

 1.57894736842105E-04

 6.8421052631579E-04

 6.8421052631579E-04

 1.05263157894737E-04

 1.05263157894737E-04

 6.31578947368421E-04

 6.31578947368421E-04

 7.84736842105263E-02

 7.84736842105263E-02

 2.48421052631579E-02

 8.10526315789473E-03

 1.10526315789474E-03

 3.68421052631579E-04

 7.36842105263158E-04

 8.94736842105264E-04

 1.05263157894737E-04

 6.8421052631579E-04

 1.05263157894737E-04

 4.57894736842105E-03

 4.57894736842105E-03

 1.10526315789474E-03

 5.26315789473684E-04

 5.26315789473684E-05

 5.26315789473684E-04

 2.63157894736842E-04

 5.26315789473684E-05

 2.10526315789474E-04

 1.57894736842105E-04

 1.57894736842105E-04

 1.05263157894737E-04

 1.05263157894737E-04

 1.05263157894737E-04

 8.94736842105263E-04

 8.94736842105263E-04

 1.05263157894737E-04

 7.89473684210526E-04

 6.36842105263158E-03

 6.36842105263158E-03

 6.31578947368421E-03

 5.26315789473684E-05

 1.26315789473685E-03

 1.05263157894737E-04

 5.26315789473684E-05

 5.26315789473684E-05

 1.15789473684211E-03

 1.15789473684211E-03

 .004

 .004

 2.63157894736842E-04

 5.26315789473684E-05

 3.68421052631579E-03

 5.26315789473684E-04

 5.26315789473684E-04

 5.26315789473684E-04

 7.36842105263158E-04

 7.36842105263158E-04

 7.36842105263158E-04

 2.84210526315789E-03

 2.84210526315789E-03

 2.84210526315789E-03

 1.98421052631579E-02

 1.68421052631579E-03

 1.31578947368421E-03

 1.31578947368421E-03

 1.57894736842105E-04

 1.57894736842105E-04

 2.10526315789474E-04

 2.10526315789474E-04

 1.78421052631579E-02

 5.26315789473684E-05

 5.26315789473684E-05

 1.75789473684211E-02

 1.75789473684211E-02

 2.10526315789474E-04

 2.10526315789474E-04

 3.15789473684211E-04

 3.15789473684211E-04

 3.15789473684211E-04

 1.33357894736842

 8.42105263157894E-04

 5.26315789473684E-05

 5.26315789473684E-05

 7.89473684210526E-04

 7.89473684210526E-04

 1.33247368421053

 1.33247368421053

 .113684210526316

 1.21878947368421

 2.63157894736842E-04

 2.63157894736842E-04

 2.63157894736842E-04

 4.94736842105264E-03

 4.94736842105264E-03

 3.10526315789474E-03

 3.10526315789474E-03

 1.84210526315789E-03

 6.31578947368421E-04

 4.73684210526316E-04

 1.57894736842105E-04

 3.15789473684211E-04

 2.63157894736842E-04

 4.36842105263158E-03

 4.36842105263158E-03

 4.36842105263158E-03

 4.36842105263158E-03

 7.01578947368421E-02

 7.01578947368421E-02

 7.01578947368421E-02

 7.01578947368421E-02

 1.71052631578947E-02

 1.71052631578947E-02

 1.34736842105263E-02

 1.42105263157895E-03

 8.42105263157895E-04

 5.78947368421053E-04

 6.31578947368421E-03

 5.31578947368421E-03

 5.26315789473684E-05

 0

 2.10526315789474E-04

 7.36842105263158E-04

 5.68421052631579E-03

 5.68421052631579E-03

 5.26315789473684E-05

 5.26315789473684E-05

 2.31578947368421E-03

 2.31578947368421E-03

 1.57894736842105E-03

 7.36842105263158E-04

 1.31578947368421E-03

 1.31578947368421E-03

 1.31578947368421E-03

 .352894736842105

 1.03684210526316E-02

 1.57894736842105E-04

 1.57894736842105E-04

 1.57894736842105E-04

 1.02105263157895E-02

 1.02105263157895E-02

 1.02105263157895E-02

 .224315789473684

 .131263157894737

 3.47368421052631E-02

 3.06315789473684E-02

 4.10526315789474E-03

 .096421052631579

 3.68421052631579E-04

 5.26315789473684E-05

 1.05263157894737E-04

 6.31578947368421E-04

 1.57894736842105E-04

 3.68421052631579E-04

 1.57894736842105E-04

 3.68421052631579E-04

 3.05263157894737E-03

 7.43157894736842E-02

 5.26315789473684E-05

 5.26315789473684E-05

 1.68421052631579E-03

 1.50526315789474E-02

 1.05263157894737E-04

 1.05263157894737E-04

 2.84210526315789E-03

 2.84210526315789E-03

 2.84210526315789E-03

 8.88421052631579E-02

 8.67368421052632E-02

 8.67368421052632E-02

 2.10526315789474E-03

 2.10526315789474E-03

 1.36842105263158E-03

 1.36842105263158E-03

 1.36842105263158E-03

 1.03684210526316E-02

 3.10526315789474E-03

 3.10526315789474E-03

 .003

 1.05263157894737E-04

 3.68421052631579E-04

 2.63157894736842E-04

 2.63157894736842E-04

 5.26315789473684E-05

 5.26315789473684E-05

 5.26315789473684E-05

 5.26315789473684E-05

 4.94736842105263E-03

 4.94736842105263E-03

 5.26315789473684E-05

 4.89473684210526E-03

 3.15789473684211E-04

 3.15789473684211E-04

 3.15789473684211E-04

 1.57894736842105E-03

 1.57894736842105E-03

 1.57894736842105E-03

 5.26315789473684E-05

 5.26315789473684E-05

 5.26315789473684E-05

 9.8421052631579E-03

 5.26315789473684E-04

 1.57894736842105E-04

 1.57894736842105E-04

 3.68421052631579E-04

 3.68421052631579E-04

 7.47368421052632E-03

 5.26315789473684E-05

 5.26315789473684E-05

 6.47368421052632E-03

 6.47368421052632E-03

 0

 0

 9.47368421052632E-04

 9.47368421052632E-04

 1.63157894736842E-03

 1.63157894736842E-03

 1.63157894736842E-03

 2.10526315789474E-04

 2.10526315789474E-04

 2.10526315789474E-04

 2.09473684210526E-02

 5.94736842105263E-03

 5.26315789473684E-05

 5.26315789473684E-05

 0

 5.8421052631579E-03

 4.21052631578947E-04

 5.42105263157895E-03

 5.26315789473684E-05

 5.26315789473684E-05

 1.33684210526316E-02

 1.33684210526316E-02

 1.05263157894737E-04

 1.28421052631579E-02

 1.57894736842105E-04

 2.63157894736842E-04

 2.10526315789474E-04

 2.10526315789474E-04

 2.10526315789474E-04

 1.42105263157895E-03

 1.42105263157895E-03

 1.42105263157895E-03

 7.70526315789474E-02

 7.70526315789474E-02

 7.70526315789474E-02

 7.70526315789474E-02

 .773

 .773

 .773

 .773

 .773

 8.61436842105264

 8.59726315789475

 1.36268421052632

 1.06842105263158E-02

 .003

 2.10526315789474E-04

 1.05263157894737E-04

 0

 2.68421052631579E-03

 1.68421052631579E-03

 1.68421052631579E-03

 1.42105263157895E-03

 4.21052631578947E-04

 1.05263157894737E-04

 8.94736842105263E-04

 4.57894736842105E-03

 4.57894736842105E-03

 .046

 3.68421052631579E-04

 1.57894736842105E-04

 2.10526315789474E-04

 4.11578947368421E-02

 1.05263157894737E-04

 8.42105263157895E-04

 3.63684210526316E-02

 6.31578947368421E-04

 2.10526315789474E-04

 .003

 1.47368421052632E-03

 1.15789473684211E-03

 3.15789473684211E-04

 2.15789473684211E-03

 2.15789473684211E-03

 8.42105263157895E-04

 8.42105263157895E-04

 2.92105263157895E-02

 3.05263157894737E-03

 3.15789473684211E-04

 5.26315789473684E-05

 2.68421052631579E-03

 .001

 3.68421052631579E-04

 5.26315789473684E-05

 1.05263157894737E-04

 4.73684210526316E-04

 6.84210526315789E-04

 6.31578947368421E-04

 5.26315789473684E-05

 2.31052631578947E-02

 4.21052631578947E-04

 2.63157894736842E-04

 1.57894736842105E-04

 1.86315789473684E-02

 3.63157894736842E-03

 1.57894736842105E-04

 1.57894736842105E-04

 3.15789473684211E-04

 3.15789473684211E-04

 8.42105263157895E-04

 8.42105263157895E-04

 5.26315789473684E-05

 5.26315789473684E-05

 4.71578947368421E-02

 4.51578947368421E-02

 5.26315789473684E-05

 4.51052631578947E-02

 4.73684210526315E-04

 5.26315789473684E-05

 4.21052631578947E-04

 1.57894736842105E-04

 1.57894736842105E-04

 5.26315789473684E-05

 5.26315789473684E-05

 1.57894736842105E-04

 1.57894736842105E-04

 1.15789473684211E-03

 1.15789473684211E-03

 2.13157894736842E-02

 1.05263157894737E-04

 5.26315789473684E-05

 5.26315789473684E-05

 3.26315789473684E-03

 1.57894736842105E-04

 2.52631578947368E-03

 5.78947368421053E-04

 2.10526315789473E-04

 5.26315789473684E-05

 1.57894736842105E-04

 2.36842105263157E-03

 5.26315789473684E-05

 1.52631578947368E-03

 1.57894736842105E-04

 6.31578947368421E-04

 1.46315789473684E-02

 4.63157894736842E-03

 5.26315789473684E-05

 9.94736842105263E-03

 3.15789473684211E-04

 3.15789473684211E-04

 1.57894736842105E-04

 1.57894736842105E-04

 2.63157894736842E-04

 2.63157894736842E-04

 1.10526315789474E-03

 1.10526315789474E-03

 5.26315789473684E-05

 5.26315789473684E-04

 5.26315789473684E-04

 .183473684210526

 2.63157894736842E-04

 2.63157894736842E-04

 1.68421052631579E-03

 1.15789473684211E-03

 2.10526315789474E-04

 3.15789473684211E-04

 1.57894736842105E-04

 1.57894736842105E-04

 1.73684210526316E-03

 2.10526315789474E-04

 5.26315789473684E-05

 1.47368421052632E-03

 2.52631578947369E-03

 1.05263157894737E-04

 1.57894736842105E-04

 5.26315789473684E-05

 5.26315789473684E-05

 2.15789473684211E-03

 1.05263157894737E-04

 5.26315789473684E-05

 5.26315789473684E-05

 8.94736842105263E-04

 8.94736842105263E-04

 .176052631578947

 .176052631578947

 5.26315789473684E-05

 5.26315789473684E-05

 9.84210526315791E-03

 6.31578947368421E-04

 5.26315789473684E-05

 4.21052631578947E-04

 5.26315789473684E-05

 5.26315789473684E-05

 5.26315789473684E-05

 1.05263157894737E-04

 1.05263157894737E-04

 1.42105263157895E-03

 5.26315789473684E-05

 5.26315789473684E-05

 1.05263157894737E-04

 5.26315789473684E-05

 5.26315789473684E-05

 1.10526315789474E-03

 6.52631578947369E-03

 1.15789473684211E-03

 5.26315789473684E-05

 2.10526315789474E-04

 5.10526315789474E-03

 .001

 .001

 1.05263157894737E-04

 1.05263157894737E-04

 5.26315789473684E-05

 5.26315789473684E-05

 .641684210526316

 7.10526315789473E-03

 4.73684210526316E-04

 5.26315789473684E-05

 1.31578947368421E-03

 7.89473684210526E-04

 1.68421052631579E-03

 5.26315789473684E-04

 2.26315789473684E-03

 3.68421052631579E-04

 2.63157894736842E-04

 1.05263157894737E-04

 .011

 1.36842105263158E-03

 7.36842105263158E-04

 2.63157894736842E-04

 4.05263157894737E-03

 4.57894736842105E-03

 1.05263157894737E-04

 1.05263157894737E-04

 .615105263157895

 1.36842105263158E-03

 1.57894736842105E-04

 .307157894736842

 1.15789473684211E-02

 .216578947368421

 7.82631578947369E-02

 3.68421052631579E-04

 0

 2.10526315789474E-04

 1.57894736842105E-04

 3.15789473684211E-04

 3.15789473684211E-04

 6.42105263157895E-03

 6.42105263157895E-03

 8.94736842105263E-04

 7.36842105263158E-04

 1.57894736842105E-04

 1.05263157894737E-03

 3.68421052631579E-04

 3.68421052631579E-04

 5.78947368421052E-04

 5.26315789473684E-05

 5.26315789473684E-04

 5.26315789473684E-05

 5.26315789473684E-05

 5.26315789473684E-05

 5.26315789473684E-05

 1.31578947368421E-03

 1.31578947368421E-03

 4.21052631578947E-04

 8.94736842105263E-04

 3.00526315789473E-02

 3.00526315789473E-02

 2.31052631578947E-02

 1.05263157894737E-04

 1.05263157894737E-03

 5.78947368421053E-03

 6.10526315789474E-03

 1.94736842105263E-03

 5.26315789473684E-05

 1.89473684210526E-03

 1.26315789473684E-03

 5.26315789473684E-05

 1.57894736842105E-04

 1.05263157894737E-03

 1.63157894736842E-03

 2.63157894736842E-04

 .001

 3.68421052631579E-04

 8.94736842105263E-04

 5.26315789473684E-05

 8.42105263157895E-04

 3.68421052631579E-04

 3.68421052631579E-04

 6.42105263157894E-03

 6.42105263157894E-03

 1.05263157894737E-04

 1.36842105263158E-03

 2.63157894736842E-04

 1.05263157894737E-04

 5.26315789473684E-05

 2.89473684210526E-03

 1.63157894736842E-03

 2.10526315789474E-04

 2.10526315789474E-04

 1.05263157894737E-04

 1.05263157894737E-04

 1.31578947368421E-03

 1.31578947368421E-03

 1.31578947368421E-03

 1.52631578947369E-03

 1.52631578947369E-03

 1.57894736842105E-04

 6.8421052631579E-04

 6.8421052631579E-04

 7.21052631578948E-03

 7.21052631578948E-03

 5.26315789473684E-05

 5.26315789473684E-05

 7.10526315789474E-03

 1.05263157894737E-04

 1.05263157894737E-04

 1.05263157894737E-04

 1.73684210526316E-03

 1.73684210526316E-03

 1.31578947368421E-03

 0

 4.21052631578947E-04

 .127947368421053

 9.29473684210526E-02

 5.26315789473684E-05

 5.26315789473684E-05

 9.28421052631579E-02

 5.78947368421052E-04

 5.26315789473684E-04

 5.26315789473684E-05

 2.36842105263158E-02

 1.15789473684211E-03

 5.78947368421053E-04

 1.57894736842105E-04

 1.57368421052632E-02

 1.05263157894737E-03

 3.15789473684211E-04

 6.8421052631579E-04

 1.05263157894737E-04

 3.89473684210526E-03

 1.07368421052632E-02

 1.07368421052632E-02

 2.68421052631578E-03

 5.26315789473684E-05

 5.26315789473684E-05

 5.26315789473684E-05

 5.26315789473684E-05

 5.26315789473684E-04

 1.57894736842105E-04

 3.68421052631579E-04

 1.78947368421052E-03

 1.52631578947368E-03

 2.63157894736842E-04

 1.57894736842105E-04

 1.57894736842105E-04

 5.26315789473684E-05

 5.26315789473684E-05

 5.26315789473684E-05

 5.26315789473684E-05

 7.57894736842105E-03

 5.26315789473684E-05

 5.26315789473684E-05

 2.63157894736842E-04

 1.57894736842105E-04

 5.26315789473684E-05

 5.26315789473684E-05

 3.15789473684211E-04

 3.15789473684211E-04

 6.94736842105263E-03

 6.94736842105263E-03

 8.57894736842105E-03

 8.57894736842105E-03

 1.05263157894737E-04

 2.63157894736842E-04

 4.21052631578947E-04

 1.05263157894737E-04

 5.26315789473684E-05

 1.57894736842105E-04

 1.05263157894737E-04

 1.57894736842105E-04

 1.05263157894737E-04

 1.05263157894737E-04

 5.26315789473684E-05

 6.94736842105263E-03

 2.94736842105263E-03

 1.68421052631579E-03

 1.05263157894737E-04

 1.57894736842105E-04

 6.8421052631579E-04

 5.26315789473684E-05

 6.8421052631579E-04

 1.26315789473684E-03

 1.05263157894737E-04

 1.57894736842105E-04

 6.31578947368421E-04

 2.10526315789474E-04

 1.57894736842105E-04

 2.63157894736842E-04

 2.63157894736842E-04

 2.63157894736842E-04

 7.89473684210526E-03

 3.10526315789474E-03

 1.05263157894737E-04

 .003

 4.21052631578947E-04

 4.21052631578947E-04

 1.15789473684211E-03

 1.15789473684211E-03

 3.21052631578947E-03

 3.21052631578947E-03

 6.31578947368422E-04

 4.21052631578948E-04

 1.05263157894737E-04

 3.15789473684211E-04

 2.10526315789474E-04

 2.10526315789474E-04

 7.36842105263158E-04

 7.36842105263158E-04

 7.36842105263158E-04

 2.63157894736842E-04

 2.63157894736842E-04

 5.26315789473684E-05

 2.10526315789474E-04

 5.26315789473684E-04

 5.26315789473684E-04

 5.26315789473684E-04

 1.05263157894737E-04

 1.05263157894737E-04

 1.05263157894737E-04

 1.05263157894737E-04

 1.05263157894737E-04

 1.05263157894737E-04

 5.78947368421053E-04

 5.78947368421053E-04

 5.78947368421053E-04

 .152105263157895

 .152105263157895

 .152105263157895

 2.05263157894737E-03

 2.05263157894737E-03

 2.05263157894737E-03

 1.57894736842105E-04

 1.57894736842105E-04

 1.57894736842105E-04

 7.213

 7.213

 7.20142105263158

 3.14368421052632

 3.78421052631579E-02

 2.42105263157895E-03

 .077

 .312684210526316

 2.30526315789474E-02

 5.26315789473684E-05

 8.89473684210526E-03

 .264947368421053

 7.17368421052632E-02

 2.52631578947368E-03

 4.29473684210526E-02

 8.05263157894737E-03

 4.41052631578947E-02

 4.31052631578947E-02

 1.10978947368421

 1.10526315789474E-03

 1.10526315789474E-03

 1.42105263157895E-03

 1.57894736842105E-04

 4.82631578947368E-02

 5.26315789473684E-05

 .214736842105263

 .899

 1.05263157894737E-02

 1.05263157894737E-04

 .832105263157895

 5.26315789473684E-05

 5.26315789473684E-05

 2.10526315789474E-04

 2.10526315789474E-04

 1.13157894736842E-02

 1.13157894736842E-02

 2.15789473684211E-02

 2.15789473684211E-02

 2.15789473684211E-02

 2.15789473684211E-02

 2.52631578947369E-03

 2.52631578947369E-03

 2.52631578947369E-03

 2.52631578947369E-03

 2.36842105263158E-03

 1.57894736842105E-04

 4.10526315789474E-03

 4.10526315789474E-03

 3.94736842105264E-03

 3.15789473684211E-04

 3.15789473684211E-04

 3.47368421052632E-03

 3.47368421052632E-03

 1.57894736842105E-04

 1.57894736842105E-04

 1.57894736842105E-04

 1.57894736842105E-04

 1.57894736842105E-04

 2.89473684210527E-03

 2.89473684210527E-03

 2.10526315789474E-04

 2.10526315789474E-04

 2.10526315789474E-04

 6.31578947368421E-04

 6.31578947368421E-04

 4.73684210526316E-04

 1.57894736842105E-04

 2.05263157894737E-03

 2.05263157894737E-03

 2.05263157894737E-03

 1.31578947368421E-03

 1.31578947368421E-03

 1.31578947368421E-03

 1.31578947368421E-03

 1.57894736842105E-04

 5.26315789473684E-05

 1.10526315789474E-03

 6.26315789473684E-03

 6.26315789473684E-03

 6.26315789473684E-03

 6.26315789473684E-03

 6.26315789473684E-03

 .114

 4.57894736842105E-03

 4.57894736842105E-03

 4.57894736842105E-03

 2.94736842105263E-03

 2.94736842105263E-03

 5.26315789473684E-05

 0

 5.26315789473684E-05

 5.26315789473684E-04

 5.26315789473684E-05

 4.73684210526316E-04

 5.26315789473684E-04

 5.26315789473684E-04

 5.26315789473684E-04

 5.26315789473684E-04

 .107210526315789

 6.8421052631579E-03

 6.8421052631579E-03

 6.8421052631579E-03

 6.8421052631579E-03

 9.24736842105263E-02

 9.24736842105263E-02

 9.24210526315789E-02

 9.24210526315789E-02

 5.26315789473684E-05

 5.26315789473684E-05

 7.36842105263158E-04

 7.36842105263158E-04

 7.36842105263158E-04

 1.05263157894737E-04

 6.31578947368421E-04

 3.84210526315789E-03

 3.84210526315789E-03

 3.84210526315789E-03

 3.68421052631579E-04

 1.57894736842105E-03

 5.26315789473684E-05

 1.84210526315789E-03

 3.31578947368421E-03

 3.31578947368421E-03

 3.31578947368421E-03

 3.31578947368421E-03

 9.47368421052632E-04

 9.47368421052632E-04

 9.47368421052632E-04

 9.47368421052632E-04

 9.47368421052632E-04

 2.63157894736842E-04

 2.63157894736842E-04

 2.63157894736842E-04

 2.63157894736842E-04

 2.63157894736842E-04

 9.47368421052632E-04

 9.47368421052632E-04

 9.47368421052632E-04

 9.47368421052632E-04

 9.47368421052632E-04

 5.26315789473684E-05

 5.26315789473684E-05

 5.26315789473684E-05

 5.26315789473684E-05

 5.26315789473684E-05

 1.76315789473684E-02

 1.76315789473684E-02

 1.76315789473684E-02

 1.36842105263158E-03

 1.36842105263158E-03

 1.36842105263158E-03

 7.10526315789474E-03

 1.05263157894737E-04

 1.05263157894737E-04

 6.36842105263158E-03

 6.36842105263158E-03

 6.31578947368421E-04

 6.31578947368421E-04

 6.21052631578947E-03

 3.57894736842105E-03

 3.57894736842105E-03

 2.31578947368421E-03

 2.31578947368421E-03

 3.15789473684211E-04

 3.15789473684211E-04

 1.78947368421053E-03

 1.78947368421053E-03

 1.78947368421053E-03

 1.15789473684211E-03

 1.15789473684211E-03

 1.15789473684211E-03

 .187052631578947

 .172578947368421

 5.26315789473685E-04

 5.26315789473685E-04

 2.10526315789474E-04

 2.10526315789474E-04

 5.26315789473684E-05

 5.26315789473684E-05

 2.10526315789474E-04

 2.10526315789474E-04

 5.26315789473684E-05

 5.26315789473684E-05

 .172052631578947

 .172052631578947

 .172052631578947

 .172052631578947

 0

 0

 0

 0

 5.78947368421053E-04

 1.05263157894737E-04

 5.26315789473684E-05

 5.26315789473684E-05

 5.26315789473684E-05

 5.26315789473684E-05

 5.26315789473684E-05

 5.26315789473684E-05

 4.73684210526316E-04

 2.10526315789474E-04

 2.10526315789474E-04

 2.10526315789474E-04

 2.10526315789474E-04

 2.10526315789474E-04

 2.10526315789474E-04

 5.26315789473684E-05

 5.26315789473684E-05

 5.26315789473684E-05

 1.05263157894737E-04

 1.05263157894737E-04

 1.05263157894737E-04

 1.05263157894737E-04

 1.05263157894737E-04

 1.36842105263158E-02

 1.36842105263158E-02

 1.36842105263158E-02

 1.36842105263158E-02

 2.10526315789474E-04

 2.36842105263158E-03

 1.11052631578947E-02

 1.05263157894737E-04

 1.05263157894737E-04

 1.05263157894737E-04

 1.05263157894737E-04

 1.05263157894737E-04

 31.9377894736842

 .445210526315788

 .445210526315788

 .445210526315788

 5.26315789473684E-05

 5.26315789473684E-05

 2.63157894736842E-04

 2.63157894736842E-04

 3.15789473684211E-03

 5.26315789473684E-05

 .003

 1.05263157894737E-04

 .103473684210526

 3.15789473684211E-04

 1.05263157894737E-04

 1.05263157894737E-04

 7.89473684210526E-04

 3.68421052631579E-04

 5.26315789473684E-05

 1.57894736842105E-04

 5.26315789473684E-05

 1.57894736842105E-04

 5.26315789473684E-05

 6.8421052631579E-04

 .100631578947368

 6.31578947368421E-04

 3.68421052631579E-04

 2.63157894736842E-04

 1.05263157894737E-03

 1.05263157894737E-03

 1.15789473684211E-03

 1.15789473684211E-03

 8.94736842105263E-04

 6.31578947368421E-04

 2.10526315789474E-04

 5.26315789473684E-05

 .188842105263157

 6.31578947368421E-04

 .188210526315789

 .001

 8.94736842105263E-04

 1.05263157894737E-04

 1.15789473684211E-03

 2.63157894736842E-04

 8.94736842105263E-04

 6.84210526315789E-04

 5.26315789473684E-05

 2.10526315789474E-04

 4.21052631578947E-04

 3.84210526315789E-03

 3.84210526315789E-03

 1.57894736842105E-04

 1.57894736842105E-04

 5.78947368421053E-04

 5.78947368421053E-04

 .111736842105263

 .111736842105263

 1.05263157894737E-04

 5.26315789473684E-05

 5.26315789473684E-05

 2.64210526315789E-02

 2.64210526315789E-02

 30.7498421052631

 30.7498421052631

 4.00805263157895

 4.00805263157895

 7.36842105263158E-03

 1.52631578947368E-03

 7.01578947368421E-02

 5.63157894736842E-03

 2.63157894736842E-04

 4.73684210526316E-04

 9.64210526315789E-02

 7.15789473684211E-03

 0

 3.15789473684211E-04

 0

 .607842105263158

 4.73684210526316E-04

 6.36842105263158E-03

 1.57894736842105E-04

 2.73684210526316E-03

 1.36842105263158E-03

 6.50526315789474E-02

 2.06315789473684E-02

 2.91578947368421E-02

 5.68421052631579E-03

 3.15789473684211E-04

 5.25263157894737E-02

 3.62105263157895E-02

 .144105263157895

 3.71052631578947E-02

 4.44210526315789E-02

 .499578947368421

 .625947368421053

 5.26315789473684E-05

 1.639

 .174263157894737

 6.92631578947368E-02

 1.47368421052632E-03

 2.65263157894737E-02

 4.12631578947368E-02

 .105

 1.17368421052632E-02

 4.73684210526316E-04

 9.27894736842105E-02

 .831421052631579

 .615578947368421

 .615263157894737

 3.15789473684211E-04

 .173315789473684

 3.11052631578947E-02

 2.63157894736842E-03

 1.96315789473684E-02

 .068

 5.19473684210526E-02

 7.63157894736843E-03

 3.15789473684211E-04

 1.15789473684211E-03

 5.05263157894737E-03

 6.8421052631579E-04

 2.63157894736842E-04

 1.57894736842105E-04

 3.48947368421053E-02

 3.48947368421053E-02

 2.002

 .172

 2.43157894736842E-02

 .147684210526316

 1.82978947368421

 1.82978947368421

 2.10526315789474E-04

 2.10526315789474E-04

 23.2904736842105

 23.2904736842105

 8.10526315789474E-02

 4.03684210526316E-02

 2.26842105263158E-02

 2.37894736842105E-02

 1.05263157894737E-04

 3.15789473684211E-04

 2.97368421052632E-02

 17.3142631578947

 3.36842105263158E-03

 .308526315789474

 7.89473684210526E-04

 1.05263157894737E-04

 1.39473684210526E-02

 4.74736842105263E-02

 3.36842105263158E-03

 2.89473684210526E-02

 1.81484210526316

 .001

 .084

 1.31578947368421E-03

 .127631578947368

 9.89473684210526E-03

 2.68421052631579E-03

 .003

 1.54736842105263E-02

 1.88194736842105

 .362631578947368

 1.42105263157895E-03

 1.06578947368421

 6.68421052631579E-03

 6.68421052631579E-03

 6.68421052631579E-03

 .436947368421053

 .436947368421053

 .436947368421053

 .479210526315789

 .479210526315789

 5.26315789473684E-05

 5.26315789473684E-05

 5.26315789473684E-05

 4.68421052631579E-03

 4.68421052631579E-03

 4.68421052631579E-03

 2.23157894736842E-02

 1.40526315789473E-02

 1.29473684210526E-02

 1.10526315789474E-03

 1.26315789473684E-03

 6.8421052631579E-04

 5.78947368421053E-04

 2.10526315789474E-04

 2.10526315789474E-04

 6.31578947368421E-04

 1.05263157894737E-04

 5.26315789473684E-04

 5.26315789473684E-04

 4.73684210526316E-04

 5.26315789473684E-05

 5.63157894736842E-03

 5.63157894736842E-03

 2.52631578947368E-02

 3.1578947368421E-03

 1.57894736842105E-04

 5.26315789473684E-05

 2.94736842105263E-03

 6.1578947368421E-03

 1.57894736842105E-04

 5.94736842105263E-03

 5.26315789473684E-05

 8.10526315789474E-03

 5.26315789473684E-05

 7.36842105263158E-04

 7.31578947368421E-03

 2.47368421052632E-03

 1.57894736842105E-04

 3.68421052631579E-04

 1.05263157894737E-03

 8.94736842105263E-04

 2.57894736842105E-03

 2.57894736842105E-03

 1.05263157894737E-04

 1.05263157894737E-04

 0

 3.68421052631579E-04

 3.68421052631579E-04

 2.31578947368421E-03

 2.31578947368421E-03

 5.78947368421053E-04

 4.73684210526316E-04

 3.68421052631579E-04

 1.05263157894737E-04

 1.05263157894737E-04

 1.05263157894737E-04

 6.8421052631579E-04

 6.8421052631579E-04

 1.05263157894737E-04

 5.78947368421053E-04

 .415842105263158

 .03

 1.57894736842105E-04

 2.98421052631579E-02

 4.94736842105263E-03

 4.94736842105263E-03

 1.68421052631579E-02

 5.26315789473684E-05

 1.57894736842105E-03

 5.26315789473684E-05

 2.63157894736842E-04

 1.48947368421053E-02

 .341736842105263

 .339315789473684

 2.42105263157895E-03

 2.23157894736842E-02

 2.23157894736842E-02

 6.47368421052632E-03

 6.47368421052632E-03

 6.47368421052632E-03

 3.31578947368421E-03

 3.31578947368421E-03

 3.31578947368421E-03

 .263526315789474

 .263526315789474

 .263526315789474

 .263526315789474

 .263526315789474

 .216210526315789

 1.89473684210527E-03

 1.8421052631579E-03

 1.8421052631579E-03

 2.63157894736842E-04

 2.63157894736842E-04

 1.47368421052632E-03

 1.47368421052632E-03

 1.05263157894737E-04

 1.05263157894737E-04

 5.26315789473684E-05

 5.26315789473684E-05

 5.26315789473684E-05

 5.26315789473684E-05

 .143105263157895

 8.78421052631579E-02

 8.78421052631579E-02

 8.78421052631579E-02

 8.72631578947368E-02

 5.78947368421053E-04

 5.27368421052632E-02

 5.27368421052632E-02

 2.42105263157895E-02

 2.35263157894737E-02

 6.8421052631579E-04

 5.26315789473684E-05

 5.26315789473684E-05

 7.10526315789474E-03

 1.05263157894737E-04

 2.10526315789474E-04

 6.78947368421053E-03

 5.26315789473684E-05

 5.26315789473684E-05

 2.13157894736842E-02

 2.13157894736842E-02

 2.52631578947368E-03

 2.52631578947368E-03

 2.52631578947368E-03

 2.52631578947368E-03

 2.89473684210527E-02

 2.83684210526316E-02

 5.26315789473684E-04

 5.26315789473684E-04

 5.26315789473684E-04

 2.84210526315789E-03

 2.84210526315789E-03

 2.84210526315789E-03

 1.66842105263158E-02

 1.15789473684211E-03

 9.47368421052632E-04

 1.57894736842105E-04

 5.26315789473684E-05

 1.10526315789474E-03

 1.10526315789474E-03

 4.21052631578947E-04

 2.63157894736842E-04

 1.57894736842105E-04

 1.10526315789474E-03

 1.10526315789474E-03

 1.28947368421053E-02

 1.28947368421053E-02

 5.78947368421053E-04

 5.78947368421053E-04

 5.78947368421053E-04

 1.05263157894737E-04

 1.05263157894737E-04

 1.05263157894737E-04

 7.63157894736842E-03

 7.63157894736842E-03

 7.63157894736842E-03

 4.73684210526315E-04

 4.73684210526315E-04

 5.26315789473684E-05

 5.26315789473684E-05

 1.57894736842105E-04

 1.57894736842105E-04

 2.63157894736842E-04

 2.63157894736842E-04

 1.05263157894737E-04

 1.05263157894737E-04

 1.05263157894737E-04

 1.05263157894737E-04

 4.22631578947368E-02

 4.22631578947368E-02

 4.22631578947368E-02

 4.22631578947368E-02

 4.22631578947368E-02

 4.47368421052632E-03

 3.63157894736842E-03

 5.26315789473684E-04

 5.26315789473684E-04

 5.26315789473684E-04

 0

 5.26315789473684E-04

 2.94736842105263E-03

 2.94736842105263E-03

 2.94736842105263E-03

 1.57894736842105E-04

 5.26315789473684E-04

 .001

 2.10526315789474E-04

 1.05263157894737E-03

 1.57894736842105E-04

 1.57894736842105E-04

 1.57894736842105E-04

 1.57894736842105E-04

 1.05263157894737E-04

 1.05263157894737E-04

 1.05263157894737E-04

 1.05263157894737E-04

 1.05263157894737E-04

 7.36842105263158E-04

 7.36842105263158E-04

 7.36842105263158E-04

 7.36842105263158E-04

 7.36842105263158E-04

 5.26315789473684E-04

 5.26315789473684E-04

 5.26315789473684E-04

 5.26315789473684E-04

 5.26315789473684E-04

 5.26315789473684E-04

 7.94736842105263E-03

 7.94736842105263E-03

 7.94736842105263E-03

 7.94736842105263E-03

 7.94736842105263E-03

 3.68421052631579E-04

 7.57894736842105E-03

 4.21052631578947E-04

 4.21052631578947E-04

 4.21052631578947E-04

 1.57894736842105E-04

 1.57894736842105E-04

 1.57894736842105E-04

 2.10526315789474E-04

 2.10526315789474E-04

 2.10526315789474E-04

 5.26315789473684E-05

 5.26315789473684E-05

 5.26315789473684E-05

 3.10526315789474E-03

 .001

 .001

 .001

 .001

 .001

 2.10526315789474E-03

 1.21052631578947E-03

 2.63157894736842E-04

 2.63157894736842E-04

 5.26315789473684E-05

 2.10526315789474E-04

 9.47368421052632E-04

 1.05263157894737E-04

 1.05263157894737E-04

 8.42105263157895E-04

 8.42105263157895E-04

 5.26315789473684E-04

 5.26315789473684E-04

 5.26315789473684E-04

 1.05263157894737E-04

 5.26315789473684E-05

 3.68421052631579E-04

 2.63157894736842E-04

 2.63157894736842E-04

 2.63157894736842E-04

 2.63157894736842E-04

 1.05263157894737E-04

 1.05263157894737E-04

 1.05263157894737E-04

 1.05263157894737E-04

 1.84210526315789E-03

 1.84210526315789E-03

 1.84210526315789E-03

 1.84210526315789E-03

 1.84210526315789E-03

 5.26315789473684E-04

 1.31578947368421E-03

 1.26842105263158E-02

 1.26842105263158E-02

 1.26842105263158E-02

 1.26842105263158E-02

 1.26842105263158E-02

 1.26842105263158E-02

 1.07894736842105E-02

 1.07894736842105E-02

 8.47368421052632E-03

 8.47368421052632E-03

 8.47368421052632E-03

 1.57894736842105E-04

 1.57894736842105E-04

 8.15789473684211E-03

 2.31578947368421E-03

 2.31578947368421E-03

 2.10526315789474E-04

 2.10526315789474E-04

 2.10526315789474E-03

 1.05263157894737E-04

 .002

 1.15789473684211E-03

 1.15789473684211E-03

 1.15789473684211E-03

 1.15789473684211E-03

 1.15789473684211E-03

 1.15789473684211E-03

 2.89473684210526E-03

 8.42105263157894E-04

 8.42105263157894E-04

 8.42105263157894E-04

 7.89473684210526E-04

 7.89473684210526E-04

 5.26315789473684E-05

 5.26315789473684E-05

 1.36842105263158E-03

 1.36842105263158E-03

 1.36842105263158E-03

 1.36842105263158E-03

 1.36842105263158E-03

 6.8421052631579E-04

 6.8421052631579E-04

 6.8421052631579E-04

 6.8421052631579E-04

 6.8421052631579E-04

 1.13157894736842E-02

 1.13157894736842E-02

 1.13157894736842E-02

 1.13157894736842E-02

 1.47368421052632E-03

 1.57894736842105E-04

 1.57894736842105E-04

 1.15789473684211E-03

 9.47368421052632E-04

 9.47368421052632E-04

 1.26315789473684E-03

 0

 3.68421052631579E-04

 8.94736842105263E-04

 1.57894736842105E-04

 1.57894736842105E-04

 5.84210526315789E-03

 5.84210526315789E-03

 1.63157894736842E-03

 1.63157894736842E-03

 4.73684210526316E-04

 4.73684210526316E-04

 4.73684210526316E-04

 4.73684210526316E-04

 4.73684210526316E-04

 4.73684210526316E-04

 6.89473684210526E-03

 6.89473684210526E-03

 6.89473684210526E-03

 6.73684210526316E-03

 6.26315789473684E-03

 1.57894736842105E-04

 2.36842105263158E-03

 3.68421052631579E-04

 4.73684210526316E-04

 2.89473684210526E-03

 4.73684210526316E-04

 4.73684210526316E-04

 1.57894736842105E-04

 1.57894736842105E-04

 1.57894736842105E-04

 4.73684210526316E-04

 4.73684210526316E-04

 4.73684210526316E-04

 4.73684210526316E-04

 4.73684210526316E-04

 2.63157894736842E-04

 2.10526315789474E-04

 9.31052631578947E-02

 9.31052631578947E-02

 9.31052631578947E-02

 5.26315789473685E-04

 5.26315789473685E-04

 2.10526315789474E-04

 3.15789473684211E-04

 9.25789473684211E-02

 9.25789473684211E-02

 3.15789473684211E-04

 .091

 1.26315789473684E-03

 4.05263157894737E-03

 4.05263157894737E-03

 4.05263157894737E-03

 4.05263157894737E-03

 4.05263157894737E-03

 4.05263157894737E-03

 3.1578947368421E-04

 1.57894736842105E-04

 1.57894736842105E-04

 1.57894736842105E-04

 1.57894736842105E-04

 1.57894736842105E-04

 1.57894736842105E-04

 1.57894736842105E-04

 1.57894736842105E-04

 1.57894736842105E-04

 1.57894736842105E-04

 1.08947368421053E-02

 1.08947368421053E-02

 1.08947368421053E-02

 1.08947368421053E-02

 1.08947368421053E-02

 1.08947368421053E-02

 1.34968421052632

 1.34968421052632

 1.34968421052632

 1.34968421052632

 1.34968421052632

 1.34968421052632

 2.55263157894736E-02

 0

 0

 0

 0

 0

 0

 .025

 1.15789473684211E-03

 1.15789473684211E-03

 1.15789473684211E-03

 5.26315789473684E-05

 5.26315789473684E-05

 1.05263157894737E-03

 1.05263157894737E-03

 5.26315789473684E-05

 5.26315789473684E-05

 2.34210526315789E-02

 2.34210526315789E-02

 2.34210526315789E-02

 .022

 7.89473684210526E-04

 5.26315789473684E-04

 1.57894736842105E-04

 2.04210526315789E-02

 5.26315789473684E-05

 5.26315789473684E-05

 1.31578947368421E-03

 5.26315789473684E-05

 1.26315789473684E-03

 1.05263157894737E-04

 1.05263157894737E-04

 0

 0

 4.21052631578947E-04

 4.21052631578947E-04

 4.21052631578947E-04

 4.21052631578947E-04

 4.21052631578947E-04

 5.26315789473684E-04

 5.26315789473684E-04

 5.26315789473684E-04

 5.26315789473684E-04

 5.26315789473684E-04

 5.26315789473684E-04

 3.21052631578947E-03

 3.21052631578947E-03

 3.21052631578947E-03

 3.21052631578947E-03

 3.21052631578947E-03

 3.21052631578947E-03

 3.21052631578947E-03

 .121631578947368

 .121631578947368

 .121631578947368

 .121631578947368

 .121631578947368

 .121631578947368

 .121631578947368
