# Supplementary material for: Bifidobacterium Is Enriched in Gut Microbiome of Kashmiri Women with Polycystic Ovary Syndrome
Source: Genes (Basel). 2022 Feb 18;13(2):379. doi: 10.3390/genes13020379 (PMC8871983; doi:10.3390/genes13020379)
Supplement: Supplementary file 1 [file genes-13-00379-s001.zip › FigureS1B.html]

Javascript must be enabled to view this page.

magnitude

 100.001750000001

 99.8551500000006

 44.6587500000001

 13.73

 2.36465

 .2852

 .0001

 .00005

 .00005

 .2851

 .00045

 .28465

 .01545

 .0026

 .0026

 .0025

 .0012

 0

 .0013

 .01035

 .01035

 .02245

 .0144

 .0006

 0

 0

 .00495

 0

 .00005

 0

 .00005

 .00245

 .0013

 .005

 .00665

 .00665

 0

 0

 .0009

 .0009

 .0005

 .00005

 0

 .00045

 .0385

 .00025

 .00025

 .0096

 .0096

 .0041

 .0041

 .00025

 .00025

 .0243

 .00875

 .0106

 .00005

 .00085

 .0001

 .00055

 .0031

 .0003

 1.76915

 1.7474

 .00045

 .00015

 .0012

 .0038

 .00015

 .0003

 0

 .0038

 .00005

 .06625

 .10335

 .0086

 .00215

 .00085

 0

 .0037

 .0002

 .00005

 .0107

 .0004

 .00015

 .00005

 .0396

 .00035

 .00015

 0

 .00005

 .00005

 .0003

 .00275

 1.4056

 .00015

 .0004

 .0002

 .00115

 .0004

 0

 .00105

 .00015

 .00375

 .0103

 .00055

 .00015

 .00105

 .0729

 .0119

 .0008

 .00095

 .0001

 .00015

 .0099

 .00985

 .00985

 .20165

 .00055

 .00005

 .00035

 .00015

 .2006

 0

 .0007

 .00025

 .00025

 .0263

 .0001

 .01335

 .00145

 .00005

 .00045

 .01415

 .0013

 0

 .00045

 .0002

 .00025

 .0003

 0

 .00015

 .00005

 .0016

 .0001

 .0003

 .00005

 .0026

 .0002

 .00005

 .014

 .00185

 0

 .0364

 .0837

 .0005

 .0005

 .03225

 .03225

 .03225

 11.07205

 10.59105

 .0037

 .00075

 .0012

 .00175

 .0011

 .0011

 .20425

 .0007

 .197

 .00005

 .00035

 .00015

 .006

 10.2519

 .0006

 .0002

 0

 .04545

 .00055

 0

 .00005

 .00265

 .00095

 .0098

 .0001

 .0046

 .0001

 .0048

 .0003

 .0002

 .00005

 .00105

 8.7502

 .00025

 .0006

 0

 .1064

 .03995

 .08875

 .0001

 .0006

 .31495

 .01005

 .0021

 .0001

 .8664

 .00555

 .00015

 .0004

 .00025

 .00025

 0

 .0045

 .00785

 .0006

 .0072

 .00005

 .00225

 .00175

 .0005

 .00135

 .00135

 .0009

 .00055

 0

 .00035

 .0001

 .0001

 .0996

 .0996

 .0125

 .00055

 .0007

 .01125

 .0724

 .00015

 .00015

 .0019

 .00175

 .00015

 .0152

 .00035

 .0001

 .00065

 .0015

 0

 .0126

 .05325

 0

 .0001

 .0002

 .00025

 .00005

 .00015

 .0001

 .0009

 .00005

 .00085

 .0153

 .00005

 .03525

 .0001

 .0001

 .0018

 .0018

 .0068

 .00555

 .00555

 .00125

 .0009

 .00035

 .0084

 .00075

 .0007

 .00005

 .00275

 0

 .00275

 .00095

 .00055

 .00025

 .00015

 .0028

 .0001

 0

 0

 0

 .00025

 0

 .0001

 .00005

 .00025

 .0002

 0

 0

 .00185

 .00115

 .00115

 .21095

 .0182

 .00015

 .00195

 .00355

 .01255

 .00255

 .0011

 .00145

 .0153

 .00535

 .0098

 .00015

 .0008

 .0001

 .0004

 0

 .0003

 .0001

 .0001

 .1511

 .0001

 .06165

 .00015

 .0056

 .05155

 .0113

 .00015

 .0206

 .0033

 .0033

 .01915

 .01915

 0

 0

 .00045

 .00045

 .00005

 .00005

 0

 .00005

 .00355

 .00355

 .00355

 .00335

 .00335

 .00335

 .00005

 .00005

 .00005

 .17545

 .17545

 .17545

 .00295

 .00295

 .00295

 .00035

 .001

 .00075

 .00085

 .0679

 .0679

 .0679

 .06765

 .00025

 .12325

 .12325

 .12325

 .0194

 .10385

 .05055

 .05055

 .05055

 .05055

 .04865

 .04865

 .04865

 .04865

 28.3257

 26.53345

 .044

 .044

 .0001

 .00075

 .04315

 0

 0

 2.90695

 .0004

 .0004

 .3894

 .2014

 .00035

 .10805

 .0796

 .00035

 .00005

 0

 .0003

 .04075

 .00015

 .0002

 .0387

 .00075

 .00095

 1.7354

 .0001

 .00005

 .00005

 0

 .2851

 .00005

 .00005

 .00035

 0

 .07795

 .0116

 .00025

 .0035

 .015

 0

 .0049

 .0001

 .0004

 .0001

 .0464

 .13885

 .05425

 .0233

 .0028

 0

 .00235

 .00005

 .0007

 0

 .01055

 .00345

 0

 .00145

 .0013

 .00005

 .00555

 .0007

 .0103

 .0041

 .00055

 1.02915

 .00015

 0

 .00015

 .01285

 .0077

 0

 .00515

 .27875

 .00595

 .2728

 .0964

 .00055

 .0798

 .0006

 .00005

 .0028

 .0002

 .0078

 .00175

 .00015

 .0027

 .05395

 .02615

 .0134

 .0144

 .0491

 .0489

 .0002

 .0011

 .0011

 .0055

 .0005

 .005

 .24285

 .24285

 3.7008

 .01525

 .01005

 .00115

 .00405

 .00005

 .00005

 .00235

 .00185

 .0005

 .89185

 .8917

 .00015

 .03725

 .0109

 .02635

 1.07055

 .75805

 .00005

 .3031

 .0001

 .0004

 0

 .00885

 .38375

 .0218

 .0398

 .32215

 .48

 .47985

 .00015

 .02465

 .0246

 .00005

 .02565

 .0009

 .0127

 .01205

 .0003

 .0003

 .13305

 0

 0

 .05385

 .07765

 .00155

 .10465

 .10465

 .53145

 .23355

 .0178

 .0578

 .00295

 .0316

 .0001

 .0187

 .0184

 .15055

 8.8132

 .02685

 .00555

 .00765

 .01365

 .0372

 .0372

 0

 5.51055

 2.6637

 2.84685

 1.52555

 .17115

 .0383

 1.3161

 1.7048

 .0031

 .27435

 .10085

 .00335

 1.32315

 .00005

 .00005

 .0082

 .0082

 8.67045

 2.44395

 .36035

 .00285

 .04915

 .00005

 .1036

 .0042

 .1822

 1.74155

 .22355

 .15955

 .064

 .0015

 .0015

 .8394

 .06835

 .3036

 .46745

 .19755

 .12465

 .0729

 .00825

 .00685

 .0014

 .91885

 .3698

 .54905

 .1325

 .11245

 .02005

 .1319

 0

 .13185

 .00005

 2.712

 .85715

 1.85485

 .78245

 .38185

 .00285

 .39775

 .0088

 .0088

 .1442

 .1442

 .00005

 .00005

 .0105

 .0105

 .115

 .115

 .0023

 .0023

 .0023

 .23905

 .2364

 .00005

 .0031

 .23325

 .0007

 .0007

 .00055

 .00015

 .0004

 .00135

 .00135

 .00005

 .00005

 .25575

 .0001

 .00005

 .00005

 .00855

 .0002

 .0048

 .00155

 .002

 .0473

 0

 0

 .027

 .02

 .0003

 .00525

 .00525

 .004

 .0039

 0

 .0001

 .0133

 .0133

 .00595

 .00595

 .1713

 .1713

 .1116

 .11145

 .0009

 .11055

 .00015

 .00015

 .00875

 .00875

 .00875

 .0013

 .0013

 .0013

 .0008

 .0008

 .0001

 .00005

 .0004

 .00025

 .00205

 .00205

 .00205

 1.77645

 1.77645

 1.77645

 1.5256

 1.5256

 .0157

 .00045

 .01525

 .07075

 .0021

 .00005

 .0002

 .00005

 .06835

 .7141

 .59255

 .04985

 .02245

 .04925

 .0102

 .0102

 .0451

 .0037

 .0414

 0

 .175

 .175

 .47315

 .00185

 .0001

 .10055

 .04805

 .3226

 .0216

 .0216

 .0608

 .0174

 .0047

 .0047

 .0002

 .0002

 .0044

 .00215

 0

 .00225

 .00245

 .00245

 0

 0

 .0025

 .00035

 .00035

 .00025

 0

 .00155

 .00315

 .00315

 .02505

 .02505

 .00015

 .0249

 .0149

 .0149

 .0149

 .00345

 .00345

 .00345

 .09615

 .08965

 .08965

 .08965

 0

 0

 .0064

 .0064

 .0064

 .0001

 .0001

 .0001

 .01215

 .0048

 .00465

 .00465

 .0001

 .0001

 .00005

 .00005

 .00735

 .00735

 .0007

 .00005

 .00085

 .00575

 .09755

 .09755

 .09755

 .09755

 2.3579

 2.3579

 .85255

 .00005

 .00005

 .07325

 .0009

 .07235

 .26125

 .0049

 .25495

 .0014

 .51145

 .48875

 .00075

 .00045

 .0215

 .0062

 .0062

 .00035

 .00035

 1.4804

 1.47885

 .10875

 1.3701

 .00115

 .001

 .00015

 .00035

 .0002

 .00015

 .00005

 .00005

 .02495

 .02495

 .02495

 .24515

 .24515

 .24515

 .24515

 .24515

 .1211

 .1211

 .06255

 .06255

 .0553

 0

 .0165

 .00195

 .00465

 .01225

 .01995

 .00715

 .00005

 .00005

 0

 .0057

 .00135

 .0001

 .0001

 .0011

 .0011

 .0011

 0

 .0002

 .0009

 .0134

 .0134

 .0134

 .0134

 .04065

 .04065

 .04065

 .00095

 .00005

 .001

 0

 .0001

 .0027

 .0003

 .00275

 .00015

 .03265

 0

 0

 .0034

 .0034

 .0034

 .0034

 13.0865

 1.29855

 1.2038

 .7115

 .00045

 .00005

 .00005

 .0002

 .00005

 .0001

 .00005

 .00005

 .71065

 0

 .05505

 .0002

 .17995

 .47545

 .00005

 .00005

 .0003

 .0003

 .3483

 .00725

 .00005

 .00105

 .0028

 .0002

 .001

 .0011

 .00105

 .00155

 .00055

 .001

 .0016

 .0004

 .0002

 .001

 .005

 .00385

 .00005

 .0011

 .09155

 .01525

 .0741

 .0022

 .00145

 .00145

 .0022

 .0022

 .0096

 .0096

 .0001

 0

 .0001

 .0014

 0

 .0014

 .0004

 .0002

 .0002

 .0005

 .0005

 .001

 0

 .001

 .08105

 .08105

 0

 0

 0

 0

 .0704

 .0018

 .06855

 .00005

 .0005

 .0005

 .00015

 .00015

 .00015

 .00015

 .0023

 .0023

 .0011

 .0011

 .0644

 .0644

 .00465

 .0046

 .00005

 0

 .01505

 .00935

 .0044

 .00005

 .0017

 .00005

 .00065

 .0025

 .00295

 .00295

 0

 .00265

 .00265

 .0001

 .0001

 .0733

 .00005

 .00005

 .0105

 .00065

 .00985

 0

 .01705

 .0112

 .00585

 .0127

 .0039

 .00875

 .00005

 .0243

 .00615

 .00005

 .0127

 .0054

 .00005

 .00005

 .00165

 .00165

 .007

 .007

 .05565

 .05565

 .05565

 .0031

 .0031

 .0001

 .0001

 .00005

 .00005

 .00005

 .00005

 .00045

 .00005

 0

 .00005

 .00025

 .0001

 .00005

 .00005

 .0002

 .0002

 .002

 .002

 .0002

 .0002

 .02315

 .02315

 .00015

 .0001

 .00005

 .00065

 .00065

 .0007

 .0004

 .0003

 .00345

 .0017

 .0002

 .00155

 .00055

 .00055

 .00205

 .0015

 0

 .0001

 .00045

 .0046

 .0046

 0

 0

 .00145

 .00145

 .00955

 .00955

 .00285

 .00285

 .00285

 .00285

 .01475

 .01475

 .0094

 .00025

 .00795

 .0012

 .00535

 .00025

 .0035

 .0016

 0

 0

 .00005

 .00005

 .00005

 .00005

 .0014

 .0014

 .0014

 .00065

 .0004

 .00035

 .04945

 .04945

 .04945

 .04945

 4.13105

 .15385

 .01565

 .00115

 0

 .00115

 .0013

 .0013

 .00005

 .00005

 0

 0

 .00005

 .00005

 .0099

 0

 .0002

 .0001

 .00485

 .0043

 .00005

 .0004

 .00005

 .00005

 .00315

 .00315

 .118

 .0775

 .071

 .0006

 .0059

 .0003

 .0003

 .00005

 .00005

 .0015

 .0015

 .01975

 0

 0

 .001

 .01875

 .00275

 .00235

 .0004

 .00005

 .00005

 .00805

 .00805

 .00805

 .00805

 .0202

 .0202

 .0202

 3.33505

 .153

 .0585

 .0022

 .00055

 .0339

 .0089

 .01295

 .00795

 .00435

 .00045

 .00315

 .0001

 .0001

 .01535

 .0007

 .00135

 .0001

 .00295

 .0033

 .00695

 .0517

 .0028

 .0005

 .0484

 .00105

 .00025

 .0002

 .0006

 .01835

 .01835

 2.69815

 .0042

 .0005

 .0012

 .0025

 .0222

 .00005

 .00115

 .0011

 .00145

 .00095

 .0175

 2.66485

 .19465

 .1036

 .0008

 .02335

 2.34245

 .00515

 .00105

 .0041

 .00175

 .00175

 .02195

 .00625

 .0001

 .0001

 .00605

 .00025

 .00015

 .0001

 .01245

 .0007

 .01175

 .0005

 .0005

 .00005

 .00005

 .00245

 .00245

 .00215

 .00215

 0

 .00215

 .01035

 .01035

 0

 .0089

 .00095

 .0005

 .00045

 .00015

 .00015

 .00005

 .00005

 .00025

 .00025

 .16845

 .0342

 .00225

 .00075

 .0007

 .0002

 .0031

 .0011

 .0261

 .0037

 .001

 .0002

 .0015

 .001

 .1247

 .1247

 .0046

 .0046

 .00125

 .00125

 .01025

 .00485

 .00005

 0

 .00005

 .0002

 .00005

 .00135

 .00315

 .0014

 .0014

 0

 0

 .00005

 .00005

 .0033

 .00005

 .0001

 .00315

 .00065

 .00065

 .00855

 .00855

 .00335

 .00005

 0

 .00515

 .0006

 .0006

 .00025

 .00035

 .10115

 .10115

 .002

 .0001

 .00065

 .0001

 .001

 .04125

 .05605

 .0016

 .0016

 .0016

 .1584

 .1584

 .1584

 .3414

 .33645

 .0057

 .0011

 .00295

 .00005

 .0016

 .0012

 .0002

 .001

 .0011

 .0011

 .1761

 .0001

 .0015

 .0317

 .00045

 .00005

 .00215

 .0002

 .0002

 .00005

 .1397

 .0209

 .0001

 .0005

 .0203

 .095

 .0928

 .0001

 .0021

 .00155

 .00005

 .0015

 .00905

 .00235

 .00215

 .00455

 .0149

 0

 .0148

 0

 .0001

 .0001

 .0001

 .01085

 .01085

 .00295

 .0011

 .00005

 0

 .00105

 .00185

 .00185

 .002

 .002

 .002

 .0074

 .0074

 .0006

 .00055

 .00005

 .00075

 .0001

 .00065

 .0006

 0

 .00055

 .00005

 .00005

 .00005

 .0046

 .0001

 .00045

 .00405

 0

 0

 .0008

 .0008

 .09485

 .01495

 .01185

 .01185

 .0031

 .0031

 .0797

 .0797

 .0071

 .0001

 .0725

 .0002

 .0002

 .0002

 .16385

 .00215

 .002

 .002

 .00015

 .00005

 .0001

 .1614

 .0063

 .00135

 .00495

 .0026

 .00035

 .00025

 .0003

 .0001

 .0016

 .00635

 .00185

 .001

 0

 0

 .00015

 .00335

 .13755

 .00025

 .00245

 0

 .01945

 .00225

 .0001

 .00085

 0

 .00005

 .00005

 .001

 .00035

 .001

 .00005

 .00645

 0

 .00145

 .00015

 .00175

 .00005

 .0106

 .08925

 .00015

 .00005

 .00005

 .00005

 .00845

 .00845

 .0003

 .0003

 .0003

 .0012

 .0012

 .0012

 0

 .0012

 .03345

 .03345

 .03345

 .03345

 6.2831

 .07975

 .018

 .01255

 .0001

 .0005

 0

 0

 .00025

 .00015

 .001

 .00015

 .00545

 .00025

 .00005

 .0001

 .0001

 .0012

 .00035

 .0029

 .0032

 .00045

 .00275

 .0002

 .0002

 .002

 .00005

 .0002

 .00175

 0

 0

 .00005

 .00005

 .06175

 .0001

 0

 .0001

 .0616

 .0001

 .00005

 .00055

 .0059

 .00015

 .00015

 .0008

 .00095

 .0011

 .00005

 .0028

 .0002

 .00165

 .0023

 .0005

 .0005

 .00965

 .0001

 .0002

 .0011

 .0008

 .00125

 .0002

 .00275

 .0001

 .0001

 .0005

 .00045

 .0002

 0

 .0003

 .00025

 .00115

 .0001

 .02465

 .00005

 .00005

 .08075

 .08075

 .00495

 .0001

 .0017

 .00025

 .0029

 .0002

 .0001

 .0001

 0

 .0003

 .0003

 .0012

 .0012

 0

 .00005

 .00005

 .05475

 .05415

 0

 .0006

 .00885

 .00885

 .0011

 .001

 .0001

 .00935

 .00935

 .0207

 .00025

 .00025

 .00025

 .0098

 .00035

 .00035

 .0001

 .0001

 .00355

 0

 .00165

 .00005

 .00075

 .0011

 .0022

 .0022

 .0036

 .0036

 .0029

 0

 0

 .00005

 .00005

 .00115

 .00005

 .0001

 0

 0

 .00005

 .00095

 .0005

 .00005

 0

 .00045

 .0012

 .0012

 .00325

 .00325

 .0019

 .00045

 .0009

 .00005

 .00005

 .00005

 .0021

 .0021

 .0021

 .00235

 .00235

 .00235

 .0361

 .02725

 .0002

 .0002

 .00185

 .00185

 .00005

 .00005

 .0003

 .0003

 .002

 .002

 .00035

 .00005

 .0003

 0

 .0037

 .0037

 .00005

 .00005

 .01875

 .01875

 .00505

 .0005

 .00015

 .00035

 .00015

 .00015

 .00255

 .00255

 .00035

 .00035

 0

 0

 .0015

 .0015

 .00005

 .00005

 0

 .00005

 .00375

 .00375

 .00375

 .00915

 .00235

 .00005

 .00005

 .0023

 .0023

 .00175

 .00175

 .00175

 .00505

 .00505

 .0001

 .0005

 .00445

 .36505

 .0941

 .00005

 .00005

 .003

 .0025

 .0005

 0

 0

 0

 .00335

 .00335

 0

 .003

 .0006

 0

 .00215

 .00025

 .00205

 0

 .00005

 .00055

 .00145

 .0011

 .00105

 .00005

 0

 0

 .0361

 .00005

 .02235

 .00005

 .0064

 .0003

 .00015

 .0068

 0

 0

 .04535

 .04535

 .0001

 .0001

 0

 .27095

 .138

 .0011

 .1369

 .1061

 .01465

 .09145

 .02685

 .02655

 .0003

 1.49045

 1.49045

 .01175

 .00005

 .0007

 .0002

 .00025

 .01055

 .0001

 .0001

 .0059

 .002

 .00045

 .00345

 .0008

 .00025

 .0005

 .00005

 .0001

 .0001

 .08645

 .0037

 .00045

 .00995

 .0071

 .00005

 .00005

 .00005

 0

 .04165

 .00075

 .00195

 .00105

 .0197

 .0062

 .0052

 0

 .00025

 .00005

 0

 .0004

 .00005

 .00025

 1.0302

 .43215

 .0135

 .58455

 .0756

 .01095

 .00095

 .0099

 .0286

 .0252

 .00005

 .00005

 .11705

 .11585

 .0012

 .0013

 .0003

 .00065

 .00035

 .0002

 .0002

 .00085

 .00085

 .00045

 .00045

 .0004

 .0004

 0

 0

 .0012

 .0012

 .15185

 .15185

 .0491

 .0113

 .0009

 0

 .00015

 .00075

 .0022

 .00005

 .00045

 .0017

 .00725

 .00725

 .0006

 .00025

 .00035

 .0002

 .0001

 .00005

 .00005

 .00015

 .00015

 .0002

 .0002

 0

 .0002

 .0197

 .0197

 .00005

 .0196

 .00005

 .00125

 .00015

 .00005

 .0001

 .0011

 .0011

 .0061

 .0061

 .00015

 0

 0

 .0002

 .00575

 .0018

 .0018

 .0018

 .00045

 0

 0

 .00045

 .00045

 .00035

 .00035

 .00035

 .00795

 .00795

 .00795

 .00015

 .00015

 .00015

 .00015

 0

 .01305

 .00365

 .0025

 .0025

 0

 0

 .00115

 .00115

 0

 0

 .0085

 .00055

 .00055

 .00765

 0

 .00765

 .0003

 .0003

 .0009

 .0009

 .0009

 .00675

 .00675

 .0048

 .0048

 .00005

 .00005

 .0019

 .0012

 .00015

 .0004

 .00005

 .00005

 0

 .00005

 4.01745

 4.01295

 4.01295

 .39135

 3.6216

 .00395

 .00395

 .00395

 .00055

 .00055

 .00055

 .00335

 .00335

 .00335

 .00335

 .1113

 .1113

 .1113

 .1113

 .02065

 .0206

 .0169

 .00225

 .0003

 .00195

 .01035

 .00825

 .0003

 0

 .00015

 .0001

 .00155

 .0043

 .0043

 .00285

 .00285

 .00205

 .0008

 .00085

 .00085

 .00085

 .00005

 .00005

 .00005

 .00005

 .40795

 .0128

 .01275

 .01275

 .01275

 0

 .00005

 .00005

 .00005

 .2488

 .19035

 .08095

 .0808

 .00015

 .1091

 .0001

 .0001

 0

 .00005

 .00015

 .0001

 0

 .00025

 .00015

 .00045

 .0981

 .00005

 0

 .0009

 .00005

 .00865

 .0003

 .0003

 .0035

 .0035

 .0035

 .05355

 .0513

 .0513

 .0002

 .0002

 .00205

 .00205

 0

 0

 .0014

 .0014

 .0014

 .0084

 .00365

 .00365

 0

 .0036

 .00005

 .00255

 .00255

 .00005

 .0001

 .0024

 .00035

 .00035

 .00005

 .0001

 .0002

 .0001

 .00005

 0

 .00005

 .00005

 .00005

 .0017

 .00165

 .00165

 .00005

 .00005

 .00005

 .00005

 .00005

 .01635

 .00145

 .00015

 .00015

 .0013

 .0013

 .0102

 .0001

 .0001

 .00065

 0

 .00005

 .0006

 .00765

 .00765

 .0018

 .0018

 .00465

 .00465

 .00465

 .00005

 .00005

 .00005

 .02125

 .008

 .00035

 .00005

 .0003

 .0076

 .0002

 .0074

 .00005

 .00005

 .0033

 .0033

 .00255

 .00005

 .0007

 .0012

 .0012

 .0012

 .00875

 .00875

 .00875

 .10035

 .10035

 .10035

 .10035

 .9452

 .9452

 .9452

 .9452

 .9452

 3.8247

 3.80305

 .927349999999999

 .0099

 .0013

 .0005

 .00045

 .00035

 .00075

 .00075

 .00145

 .0001

 .0007

 .00025

 .0004

 .0064

 .0064

 .0529

 .00015

 .00015

 .0501

 .0004

 .00005

 .04175

 .0013

 .0005

 .0061

 .00105

 .00075

 .0003

 0

 .0001

 .00005

 .00005

 .00055

 .00055

 .00085

 .00085

 .0001

 .00005

 .00005

 .0261

 .00165

 .00065

 .0001

 .0009

 .0002

 .0001

 0

 .0001

 .00225

 .00225

 .0197

 .00005

 .0002

 .00005

 .0001

 .01545

 .00385

 .0002

 .0002

 .00155

 .00155

 .00055

 .00055

 .01785

 .0165

 .01645

 .00005

 .0002

 .0002

 0

 .00005

 .00005

 0

 0

 0

 .0001

 .0001

 0

 0

 .001

 .001

 .0122

 .0001

 0

 .0001

 .00505

 0

 .0002

 .00475

 .0001

 .00015

 0

 .00015

 .00115

 0

 .00085

 0

 .0003

 .00515

 .0047

 0

 .00045

 .0005

 .0005

 .0001

 .0001

 .0007

 .0007

 .0001

 .0004

 .0002

 .2686

 .00015

 .00005

 0

 0

 .0001

 .00205

 .0005

 .0001

 .0005

 .00015

 .00075

 .00005

 .00005

 0

 .00005

 .00225

 .0001

 .00215

 .0022

 0

 .00005

 0

 .00005

 .00005

 .00205

 0

 0

 .00125

 .00125

 0

 .00095

 .00095

 .25965

 .25965

 .00005

 .00005

 .02185

 .00295

 .00015

 .0011

 .00065

 0

 .00105

 .0001

 .0001

 .00905

 .00005

 .00005

 .00025

 .0005

 .0004

 .00005

 0

 .00775

 .0093

 .0005

 .00005

 .00015

 .00015

 .00845

 .00035

 .00035

 .00005

 .00005

 .00005

 .00005

 .08535

 .01045

 .0001

 .002

 .0031

 .00125

 .00035

 .00005

 .00095

 .00265

 .0004

 .00015

 .0002

 0

 .00005

 .0015

 .0015

 .07125

 .0001

 .01665

 .00295

 .0357

 .01585

 .00105

 .00005

 .00095

 .00005

 .0007

 .0007

 0

 0

 .04145

 .0379

 .0379

 .00315

 .0001

 .00305

 .00005

 0

 0

 .00005

 .00035

 .00035

 .00225

 .00225

 .0006

 .00165

 .02445

 .02445

 .0186

 .0006

 .00525

 .0073

 .00385

 .00005

 .0038

 .0006

 0

 0

 .0001

 .0005

 .00265

 .0006

 .002

 .00005

 .0002

 .00015

 .00005

 .04695

 .04695

 .00055

 .0002

 .0002

 .00005

 .0001

 .00025

 .00015

 0

 0

 .00005

 .00015

 0

 .00015

 0

 .00005

 .0443

 0

 .00075

 .00005

 .00005

 .00005

 .0004

 .0004

 .00015

 0

 .00015

 .0001

 .0022

 .0022

 .0022

 .0004

 .0004

 .00025

 .00015

 .00525

 .00525

 .00005

 0

 .0052

 .0001

 .0001

 0

 .0001

 .002

 .00005

 0

 .00005

 .00195

 .0018

 .00005

 .0001

 .12615

 .10125

 0

 .10125

 .0005

 .0003

 .0002

 .01785

 .00175

 .0005

 .01155

 .00025

 0

 .00005

 .00005

 .0007

 .00005

 .00295

 .00655

 .00655

 .0005

 0

 0

 .00005

 0

 .00005

 .0003

 .0003

 .00015

 .00015

 .0113

 .0001

 .0001

 .00025

 .0002

 .00005

 .0012

 .00095

 .00005

 .0002

 .00975

 .00975

 .02245

 .02245

 0

 0

 .0014

 .00115

 0

 .00035

 .01075

 .0002

 0

 .00005

 0

 .00005

 .0007

 0

 0

 0

 .00005

 .00005

 .0077

 .0072

 .0018

 .00005

 .00005

 0

 .00055

 .00115

 .0054

 .0003

 0

 .00005

 .0022

 .00245

 0

 .0004

 0

 0

 .00105

 .00105

 .00105

 .01385

 .00475

 .00005

 .0047

 .00025

 .0002

 .00005

 .0034

 .0001

 .0033

 .00545

 .00545

 .00085

 .00085

 .00005

 .0008

 .00105

 .00105

 .00105

 .00075

 .00075

 .00075

 .00005

 .00005

 .00005

 .00105

 .00105

 .00105

 .11145

 .11145

 .11145

 .00015

 .00015

 .0001

 .00005

 .00125

 .00125

 .00125

 2.8634

 2.8634

 2.85725

 1.17515

 .0065

 .0003

 .0215

 .0698

 .0108

 0

 .00035

 .28515

 .02355

 .0012

 .0058

 .0115

 .0205

 .04145

 .3098

 .00035

 .0004

 .00125

 .00035

 .00695

 .00005

 .0574

 .34195

 .00255

 0

 .46265

 .0002

 .0002

 .0001

 .0001

 .00585

 .00585

 .0123

 .0123

 .0123

 .0123

 .0045

 .0045

 .0033

 .0033

 .0001

 .0032

 0

 0

 0

 .0012

 .0012

 .0012

 0

 .0032

 .0032

 .0032

 .0032

 .003

 .0002

 .00375

 .00375

 .00375

 .00095

 .00095

 .00255

 .00255

 .00025

 .00025

 0

 0

 0

 .00395

 .00395

 .00395

 .00395

 .0001

 .00385

 .00625

 .00625

 .00625

 .00625

 .00625

 35.7674

 .50115

 .50115

 .50115

 .00005

 .00005

 .00045

 .00045

 .00065

 .00065

 .0059

 0

 .00115

 .003

 .00175

 .10325

 .00015

 .0001

 .00105

 .0003

 .00025

 .00025

 .00015

 .00015

 .0011

 .0004

 .00005

 .0993

 .00135

 .0002

 0

 .00115

 .0045

 .00005

 .00445

 .0034

 .0034

 .00125

 .00125

 .0004

 .0004

 0

 .21945

 .0018

 .21765

 .0005

 .0005

 .00125

 .00105

 .0002

 .00155

 .0002

 .00135

 .0001

 .0001

 .0005

 .0005

 .11345

 .11345

 .0001

 .00005

 .00005

 0

 0

 .04305

 .04305

 34.03805

 34.03805

 3.4877

 3.4877

 .0043

 .0031

 .01445

 .00005

 0

 .1151

 .0347

 .0013

 .19995

 .00065

 .00705

 .00005

 .0001

 .0018

 .09545

 .0118

 .00035

 .038

 .0131

 .00045

 .01695

 .02355

 .0072

 .02705

 .1129

 .28645

 .7696

 .0002

 1.70205

 .15795

 .07365

 .00245

 .0521

 .0191

 .0843

 .0128

 .00055

 .07095

 .40685

 0

 0

 .163

 .163

 .2074

 .01735

 .0023

 .00005

 .0342

 .04625

 .10725

 .00685

 0

 .0013

 .00435

 .00105

 .00015

 0

 .0296

 .0296

 2.27135

 .02955

 .0159

 0

 .01365

 2.24155

 2.24155

 .00025

 .00025

 27.2451

 27.2451

 .1769

 .0497

 .01275

 .0159

 .0012

 .0249

 21.4713

 .0004

 .2996

 0

 .00075

 .00005

 .01

 .0722

 .0045

 .0135

 .5215

 .00225

 .05525

 .002

 .167

 .0084

 .00275

 .00285

 .00975

 2.8223

 .3777

 .0044

 1.1153

 .00105

 .00105

 .00105

 .46805

 .46805

 .46805

 .9403

 .9403

 .00005

 .00005

 .00005

 .0041

 .0041

 .0041

 .316

 .2463

 .2249

 .00005

 .0123

 .00905

 .0091

 .0065

 .0026

 .00015

 .00015

 .0013

 .00115

 .00005

 .0001

 .00215

 .00175

 .0003

 .0001

 .057

 .057

 .05285

 .003

 .00005

 .00295

 .0305

 .0001

 .0304

 .00925

 .00005

 .00015

 0

 0

 .00055

 .0085

 .0023

 0

 .0003

 .00145

 .00005

 .0005

 .0057

 .0057

 .00015

 0

 .00015

 .0004

 .0004

 .00155

 .00155

 .0002

 .0002

 .0002

 .0004

 .0004

 .00025

 .00015

 .00185

 .00185

 0

 .00185

 .5504

 .0148

 0

 .0148

 .0068

 .0068

 .01225

 .0005

 .00015

 .0002

 .0035

 0

 .0079

 .46685

 .46475

 .00005

 .00005

 .002

 .0497

 .0497

 .01095

 .01095

 .01095

 .0035

 .0035

 .0035

 .2879

 .2879

 .2879

 .2879

 .2879

 .11705

 .10005

 .10005

 .10005

 .0936

 .0936

 .00085

 .0008

 .00005

 .00145

 .0002

 0

 .0001

 .00115

 .0037

 .0037

 .00045

 .00045

 .01185

 .0009

 .0009

 .0009

 .0009

 .0029

 .0029

 .0029

 .0029

 .00355

 .00355

 .00355

 .0013

 .00225

 .00395

 .00395

 .00395

 .00105

 .00155

 .00135

 .00055

 .00055

 .00055

 .00055

 .00405

 .00405

 .00405

 .00405

 .00375

 .0003

 .0005

 .0005

 .0005

 .0005

 .0005

 .0006

 .0006

 .0006

 .0006

 .0006

 0

 0

 0

 0

 0

 .01395

 .01395

 .01395

 .0016

 .0016

 .0016

 .0078

 .00005

 .00005

 .00025

 .00025

 .0073

 .0073

 .0002

 .0002

 .0028

 .00275

 .00275

 .00005

 .00005

 0

 0

 .0004

 .0004

 .0004

 .00135

 .00135

 .00135

 .2141

 .19665

 .00245

 .00245

 .00035

 .00035

 .00065

 .00065

 .0009

 .0009

 .00055

 .00055

 .1942

 .1942

 .1942

 .1942

 .00065

 .00045

 .0004

 .0004

 .0004

 .00005

 .00005

 .00005

 .0002

 .0002

 .0002

 .0002

 0

 .00005

 .00005

 .00005

 .00005

 0

 .00005

 .00025

 .00025

 .00025

 .00025

 .00025

 .0164

 .0164

 .0164

 .0164

 .00035

 .00365

 .0124

 .0001

 .0001

 .0001

 .0001

 .0001

 .387

 .25615

 .05515

 .05515

 .00005

 .00005

 .0202

 .0198

 .0004

 .0001

 .0001

 .0111

 .0001

 0

 0

 .00005

 .01095

 0

 0

 .0237

 .0237

 .1977

 .1977

 .1977

 0

 .1964

 .0013

 0

 0

 .0033

 .0033

 .0033

 .0033

 .03215

 .03045

 .0006

 .0006

 .0006

 .0006

 .0006

 .0006

 .02015

 .0025

 .00135

 .0011

 .00005

 .00215

 .00215

 .00145

 0

 .00115

 .0003

 .00295

 .00295

 .0111

 .0111

 .0011

 .0011

 .0011

 .00015

 .00015

 .00015

 .00785

 .00785

 .00785

 .0001

 .0001

 0

 0

 .0001

 .0001

 0

 0

 .0016

 .0016

 .0016

 .0016

 .0394

 .0394

 .0394

 .038

 .0379

 .0001

 .0014

 .0014

 0

 0

 0

 0

 .0593

 .0593

 .0593

 .0593

 .0593

 .00545

 .0031

 .0013

 .0013

 .0013

 0

 .0013

 .00175

 .00175

 .00175

 .00005

 .0004

 .00095

 0

 .00035

 .00005

 .00005

 .00005

 .00005

 .0004

 .0004

 .0004

 .0004

 .0004

 .0019

 .0019

 .0019

 .0019

 .0019

 .00005

 .00005

 .00005

 .00005

 .00005

 .0013

 .0013

 .0013

 .0013

 .0013

 .0013

 .0051

 .0051

 .0051

 .0051

 .0051

 .00095

 .00415

 .0007

 .0007

 .0007

 0

 0

 0

 .00055

 .00055

 .00055

 .0001

 .0001

 .0001

 .00005

 .00005

 .00005

 .00815

 .00355

 .00355

 .00355

 .00355

 .00355

 .0046

 .00115

 .00115

 .00115

 .00005

 .0011

 .00035

 .00035

 .00035

 .0003

 .00005

 .0031

 .00005

 .00005

 .00005

 .00305

 .0001

 .0001

 .00295

 .00295

 .00235

 .00235

 .00235

 .00235

 .00235

 .00145

 .0009

 .0071

 .0071

 .0071

 .0071

 .0071

 .0071

 0

 .0096

 .0096

 .00595

 .00595

 .00595

 .0001

 0

 .00005

 .0058

 .00365

 .00365

 .00365

 .00005

 .0036

 .0011

 .0011

 .0011

 .0011

 .0011

 .0011

 .00265

 .00085

 .00085

 .00085

 .0006

 .0006

 .00025

 .00025

 .0009

 .0009

 .0009

 .0009

 .0009

 .0009

 .0009

 .0009

 .0009

 .0009

 .00785

 .00785

 .00785

 .00785

 .0008

 0

 .0002

 .0006

 .0015

 .0015

 .00085

 .00005

 .00025

 .00055

 .0001

 0

 .0001

 .00355

 .00355

 .00105

 .00105

 .0001

 .0001

 .0001

 .0001

 .0001

 .0001

 .00475

 .00475

 .00475

 .00415

 .00345

 0

 .00005

 .00305

 .00015

 0

 .0002

 0

 0

 .0007

 .0007

 .00035

 .00005

 .00005

 .0003

 .0003

 .00025

 .00025

 .00025

 .0003

 .0003

 .0003

 .0003

 .0003

 .0003

 0

 .08095

 .08095

 .08095

 .00115

 .00115

 .00115

 .0798

 .0798

 .00045

 .07715

 .0022

 .004

 .004

 .004

 .004

 .004

 .004

 .0003

 .0003

 .0003

 .0003

 .0003

 .0003

 0

 0

 0

 0

 0

 .29095

 .29095

 .29095

 .29095

 .29095

 .29095

 1.2319

 1.2319

 1.2319

 1.2319

 1.2319

 1.2319

 .0153

 .0001

 .0001

 .0001

 .0001

 .0001

 .0001

 0

 0

 .01495

 .01465

 .01465

 .01465

 .0127

 .0001

 .00065

 .01175

 .0002

 .0018

 .00005

 .00175

 .00005

 .00005

 .0001

 .0001

 .0002

 .0002

 .0002

 0

 0

 .00015

 .00015

 .00005

 .00005

 .0001

 .0001

 .0001

 .0001

 .0001

 .00025

 .00025

 .00025

 .00025

 .00025

 .00025

 .002

 .002

 .002

 .002

 .002

 .002

 .002

 .1293

 .1293

 .1293

 .1293

 .1293

 .1293

 .1293
