# Supplementary material for: Bifidobacterium Is Enriched in Gut Microbiome of Kashmiri Women with Polycystic Ovary Syndrome
Source: Genes (Basel). 2022 Feb 18;13(2):379. doi: 10.3390/genes13020379 (PMC8871983; doi:10.3390/genes13020379)
Supplement: Supplementary file 1 [file genes-13-00379-s001.zip › FigureS2.pdf]

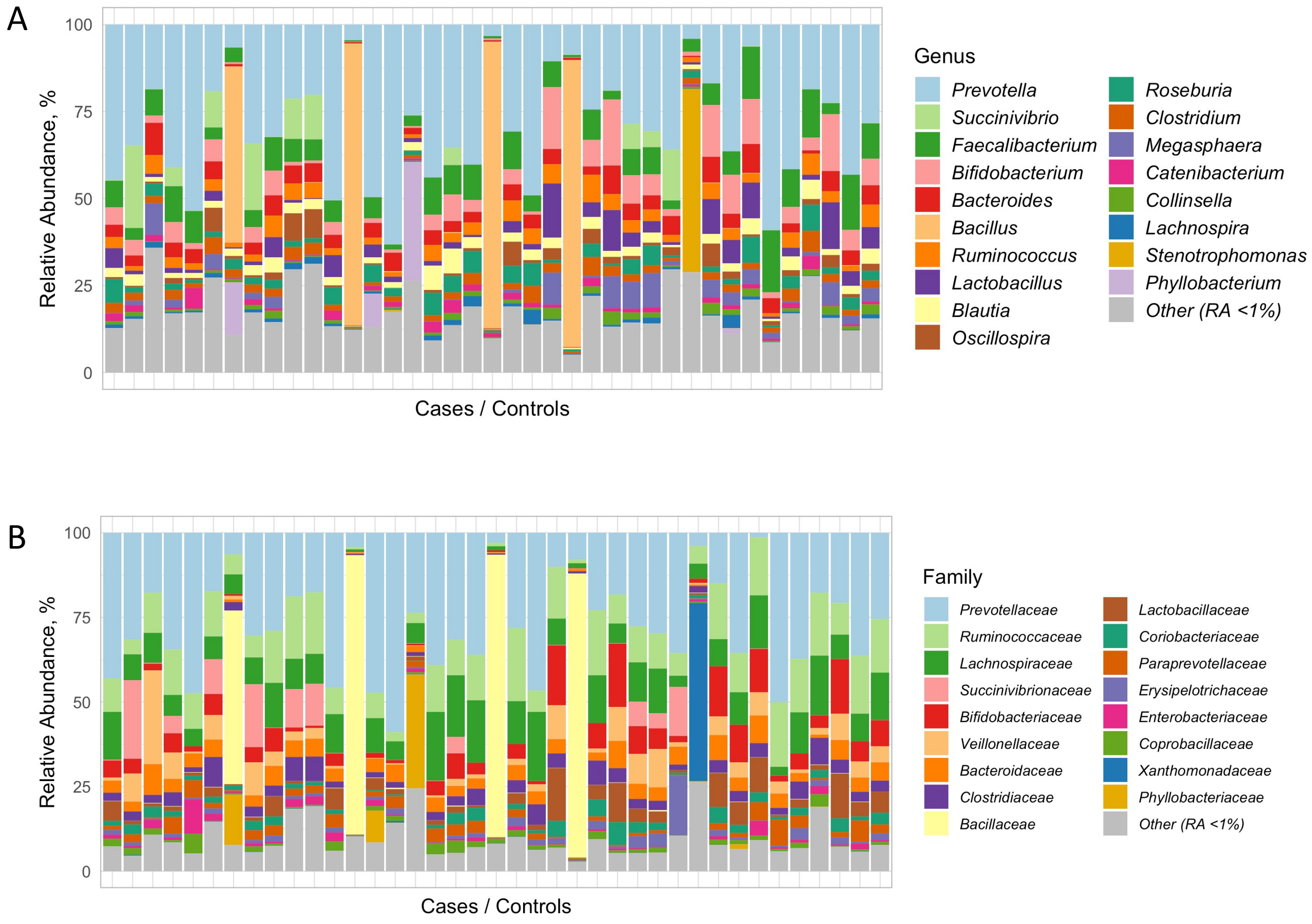

Figure S2. The relative abundancies (RA) of the OTUs at (a) genus and (b) family level for each individual. The 19 individuals with PCOS are plotted first, followed by 20 controls. For better visualisation, only OTUs with RA >1% are plotted and the less prevalent ones are grouped together in Other (RA <1%).
